# The Sap Flow Dynamics and Response of *Hedysarum scoparium* to Environmental Factors in Semiarid Northwestern China

**DOI:** 10.1371/journal.pone.0131683

**Published:** 2015-07-02

**Authors:** Jifeng Deng, Guodong Ding, Guanglei Gao, Bin Wu, Yuqing Zhang, Shugao Qin, Wenhui Fan

**Affiliations:** 1 Yanchi Research Station, School of Soil & Water Conservation, Beijing Forestry University, Beijing, 100083, P. R. China; 2 Key Laboratory of Soil and Water Conservation and Desertification Combating, Ministry of Education, Beijing Forestry University, Beijing, 100083, P. R. China; University of Vigo, SPAIN

## Abstract

*Hedysarum scoparium *is an important, fast-growing and drought-resistant shrub that has been extensively used for grassland restoration and preventing desertification in semiarid regions of northwestern China. The primary objective of this study was to investigate the diurnal and seasonal variations in stem sap flow (*J*_s_) and its relation to environmental factors. The stem heat balance method was applied to plants that were approximately 17 years old (with diameters of 25, 16, 13, and 9 mm at ground level and heights of 3.1, 1.8, 1.7 and 1.4 m) and growing under natural conditions. The vertical soil temperature profile (ST), soil surface heat flux (SoilG), volumetric soil moisture content (SWC) and meteorological variables such as solar radiation (*R*_n_), air temperature (*T*_a_), vapour pressure deficit (VPD), wind speed (*W*_s_) relative humidity (RH) and precipitation (P) were simultaneously measured at a meteorological station on site. Results indicated that *J*_s_ varied regularly during the diurnal and seasonal term. The nocturnal *J*_s_ was substantial, with a seasonal variation similar to the patterns of daytime *J*_s_. The magnitude of *J*_s_ changed considerably between sunny and rainy days. Redundancy (RDA) and Kendall’s tau analysis suggested that daily *J*_s_ in large plants was more sensitive to environmental factors, and the variation in daily *J*_s_ during the growing season could be described by a multiple linear regression against environmental variables including *T*_a_, VPD, *W*_s_, RH, ST, and SoilG. While the nocturnal *J*_s_ in smaller plants was more sensitive to meteorological factors. *T*_a_, VPD, and *W*_s_ were significantly correlated with nighttime *J*_s_. The hourly nighttime sap flow rate of *H*. *scoparium* corresponded closely to *T*_a_ and VPD following a non-linear pattern. The results of this study can be used to estimate the transpiration of *H*. *scoparium*.

## Introduction

China is among the countries most severely affected by desertification. In the early 1950’s, more than 70 severe sandstorms occurred which led to topsoil losses, affecting the north central plain and northwestern China and eventually encroaching on the living environment of local people. In order to deal with problems associated with desertification and sandstorms, many ecological engineering projects have been established to minimize the impacts of desertification [1].

*Hedysarum scoparium* (Fisch. & C.A. May) *(H*. *scoparium*) is a vivacious leguminous and deciduous shrub growing in arid and semi-arid regions of northern China. The shrub can reach a height of 0.8–3.0 m. The extensive root system of the large shrub can spread to 10 m horizontally and penetrate soil to 5–8 m depth (15 m in some cases). The extensive root system allows *H*. *scoparium* to survive on sandy desert soils (water content below 3%) and tolerate extremely high temperatures (from 50–60°C) and dry climates (< 200 mm annual precipitation). Due to its high tolerance to drought and extreme temperatures, *H*. *scoparium* is an ideal xerophyte shrub for resisting desertification, and preventing soil, wind, and water erosion by providing sand dune stabilization.

In 1956 the *Hedysarum scoparium* shrub was used to establish a vegetation belt along rail lines from Lanzhou to Baotou, which runs along the edge of the Tengger Desert [1]. Designed purpose for the vegetation belt was to stabilize migrating desert dunes in the Shapotou area at the Tengger Desert southeastern edge. This project is viewed as a successful model for desertification control and ecological restoration along the railway infrastructure in the arid desert region in China.

Restoration of desert ecosystems using xerophyte shrubs produces a wide range of hydrological effects [1]. There are more apparent in semiarid regions due to the large temporal and spatial variability in precipitation. However, our understanding of the water relations and physiological responses to environmental factors of these desert-living shrubs is limited, and a better understanding of transpiration by desert plants is urgently required. At this time, few studies have been conducted to quantify the diurnal and seasonal transpiration of *H*. *scoparium* under natural conditions.

In recent years, various methods using dyes, radioisotope, tracers, lysimeters, a heat balance and thermal diffusion have been used to measure plant transpiration [2]. However, many of these measurement techniques are invasive and may damage the plant. The stem heat balance technique has been widely used for transpiration studies because it maintains the basic conditions of normal tree growth allows continuous monitoring over a period of time, is relatively easy, does not induce any modification of the environment, and is non-destructive to the shrub [3–8]. Many studies have used the stem heat balance method to measure the fluid-flow characteristics of various plants [2, 3]. Moreover, shrubs with low stem porosity such as *H*. *scoparium*, are suitable for the application of the stem heat balance technique. For these reasons, we used the method in this study.

Eco-hydrological studies of transpiration indicate that plants regulate sap flow (*J*_s_) by adjusting stomatal conductance in response to changes in environmental variables, such as radiation intensity, soil moisture, rainfall, air temperature (*T*_a_), and wind speed (*W*_s_) [9]. Previous studies have focused mainly on the effect of individual environmental factors on plant physiology [10]. However, the interaction of exterior environmental factors should not be neglected, as a certain combination of environmental factors likely play a role in determining plant respiration. For example, semiarid regions of northwestern China are characterized by summers with long water deficit periods and high solar radiation (*R*_n_); *T*_a_, and vapour pressure deficit (VPD). These variables are correlated with each other, potentially with a compensation effect that influences plant growth under adverse environmental conditions. Plants may have acquired certain genetic characteristics enabling them to dynamically respond to different combinations of environmental factors. As a dominant native species, *H*. *scoparium* has adapted to survive in a variety of harsh environmental conditions by adjusting its transpiration [10, 11].

The measurement of *J*_s_ can provide an accurate estimation of actual plant water consumption, but is often influenced by environmental factors. As a result, accurately estimating the sap flow rate and exploiting its relationships with various environmental factors or their interactions is important not only for physiological research but also for the appropriate management of this plant to combat desertification in arid regions. The objectives of this study are to i) determine the diurnal and seasonal sap flow dynamics; ii) analyze the characteristics of daytime and nighttime sap flow and their environment dependence by using regression and redundancy analysis (RDA) methods. Our results will provide the useful information for supporting the management of this ecologically important shrub in semiarid Northwestern China.

## Materials and Methods

### Experimental site and plant material

Beijing Forestry University is responsible for the study site. The study site does not contain any national park or other protected area of land or sea. Environment Protection and Forestry Bureau of Yanchi County supervised the protection of wildlife and environment. The location is not privately owned or protected, and the field studies did not involve endangered or protected species. No specific permits were required for the described field studies. For Yanchi Research Station was found by Beijing Forestry University and authorized by China government. The authorities and we authors confirm that the field studies did not involve endangered or protected species.

**Fig 1:**
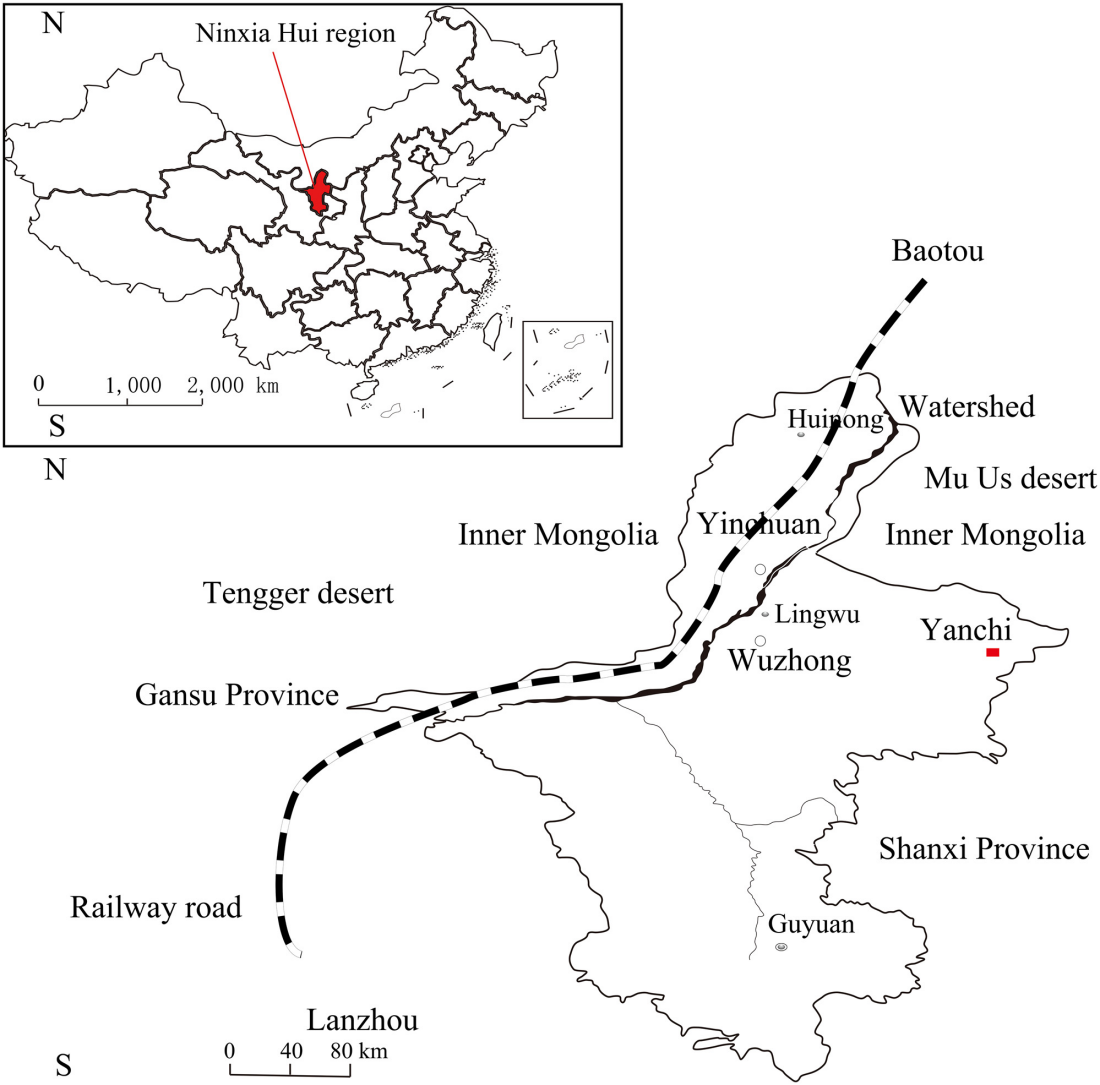
A map of the study site and its location in China.

The experimental area was located in Ningxia Yanchi Research Station of State Forestry Administration (between 37°04´N and 38°10´N, and between 106°300´E and 107°410´E, with altitude 1354 m above sea level) (Fig 1), covering an area of approximately 8661.3 km^2^. The climate is dominated by a semiarid continental monsoon of the mid-temperate zone, with long winters, short summers, a late spring, and an early autumn. The annual precipitation averages 287 mm (1950~2010). About 70% of the total precipitation occurred from July to September. Mean annual potential evaporation is 1273 mm. A biologically active temperature accumulation (>0°C) is 2810°C, with a mean annual temperature of around 8.1°C, with lowest monthly mean temperatures being -24.2°C in January and high monthly temperature of 34.9°C in July. The prevailing wind is mainly from the northwest, and wind speed averages 2.6 m.s^-1^. The landscape is a typical transitional zone, the terrain changes from the Loess to the Ordos plateau. Soils are primarily dark loessial soil, eolian sandy soil and sierozem soil, with some loess deposits, saline soil, planosol, along with other soil types. Vegetation type varies from dry steppe to desert grassland.

*H*. *scoparium* grows naturally in the study area. It is a deciduous desert shrub with small leaves, and has also been confirmed to be a C_3_ plant. The phenological character of *H*. *scoparium* is clearly indicative of an adaptive function to the local environment. *H*. *scoparium* has bud-burst at the end of April, blooms in May, is fructified in June, becomes mature in June, and begins to wither and turn yellowish in the middle of October (S1 Fig).

During our research, a 5× 5 m plot was selected at the study site. Four plants within the plot were carefully selected for monitoring based on their stem diameter. The basic characters of the plant, such as height and coverage, were also investigated (The plant stem diameters were 26, 19, 15 and 11 mm; the plant heights were 3.1, 1.8, 1.7, and 1.4 m; and the crown projected areas were 1.5 × 2.0, 1.0 × 1.2, 0.8 × 1.2 and 0.8 × 0.8 m, respectively).

### Field set up of the sap flow measurement system

Model SGB25, SGB16, SGB13, and SGB9 gauges (Flow32 meters, Dynamax Inc., Houston, TX, USA) were mounted at the stems of 17 year old *H*. *scoparium* plants during the period from 1^st^ May to 15^th^ October, 2011 (due to the changing trend of the leave area index (LAI) we measured after DOY 290, but found the sap flow rate was very low, and had almost stopped). Each sensor was installed on stem of different individual plants (S2 Fig). Data was recorded at 10 second intervals and stored as 15 minute averages using a CR1000 data logger (Campbell Scientific, Logan, UT, USA). The gauges were strictly installed following the manufacturer’s instructions [12].

Unlike other methods, Dynagages require no calibration since sap flow is directly determined by the energy balance and rates of heat convection by the sap flux (see more details at ftp://ftp.dynamax.com/Manuals/Dynagage_Manual.pdf). For stem heat balance technique will continue to give off heat to plant stem for a continuous time, its sensors are not affected by external interferences as the most common sensor types (like Trunk Heat Balance THB, Thermal Dissipation TD, Heat field deformation HFD) do. And it is different from other sap flow systems, which can get "net" sap flow data, from subtract the baseline representing the "fictitious flow" due to heat loses from the heated space. The Dynagage system is accurate, and the sap flow computations are maintained during all conditions within reasonable limits. Since the additional heat is properly accounted for in the energy balance, the user does not need any special computation to compensate for these events [12].

In our research, we used Beijing time to illustrate the diurnal and seasonal variations of sap flow rates, because the Shanxi Astronomy Observatory in Shanxi province sets the standard time (Beijing time) (GMT+08:00) in China. In this context, the local time is suitable for use (Shanxi and Ningxia provinces are in the same time zone).

### Meteorological and soil moisture measurements

Meteorological data were obtained using an on-site meteorological monitoring station (Campbell Scientific Inc., Logan, UT, USA) that has both aboveground and underground units. The above ground unit contains one CR3000, one CNR4 net radiation sensor, two PAR-LITE; one CMP3 total radiation sensor, one 034B anemometer, one HMP155a, two ombrometer sensors, six si_111 Infrared temperature sensors, measuring solar radiation (*R*_n_, W.m^-2^), photosynthetic active radiation (PAR, u.mol.s^-2^.m^-2^), vapour pressure deficit (VPD, KPa), net radiation (PAR_net, u.mol.s^-2^.m^-2^), relative humidity (RH, %), wind speed (*W*_s_, m.s^-1^), rainfall (P, mm) and air temperature (*T*_a_,°C), respectively. The underground units consisted of one ACC-SEN-SDI soil temperature humidity sensor, ten T109 and five hfp01 soil thermal throughput sensors in each pit (total of 5), which measured soil temperature (ST,°C) at depths of 10 and 30 cm and soil surface heat flux (SoilG, W.m^-2^), respectively. All variables were measured with a frequency of 10 Hz and recorded an average on every 15 minutes.

Soil moisture content (SWC, %) was obtained using a soil volumetric moisture detector (HH2 Soil Moisture Probe type ML2x and Meter type HH2) (Dynamax Inc., Houston, TX, USA) at soil depths between 0–100 cm. Soil profiles were dug every 15 days and extra measurement was taken after each rainfall event. Gaps in the data were filled using linear interpolation method. Data with obvious errors were carefully examined and removed.

**Fig 2:**
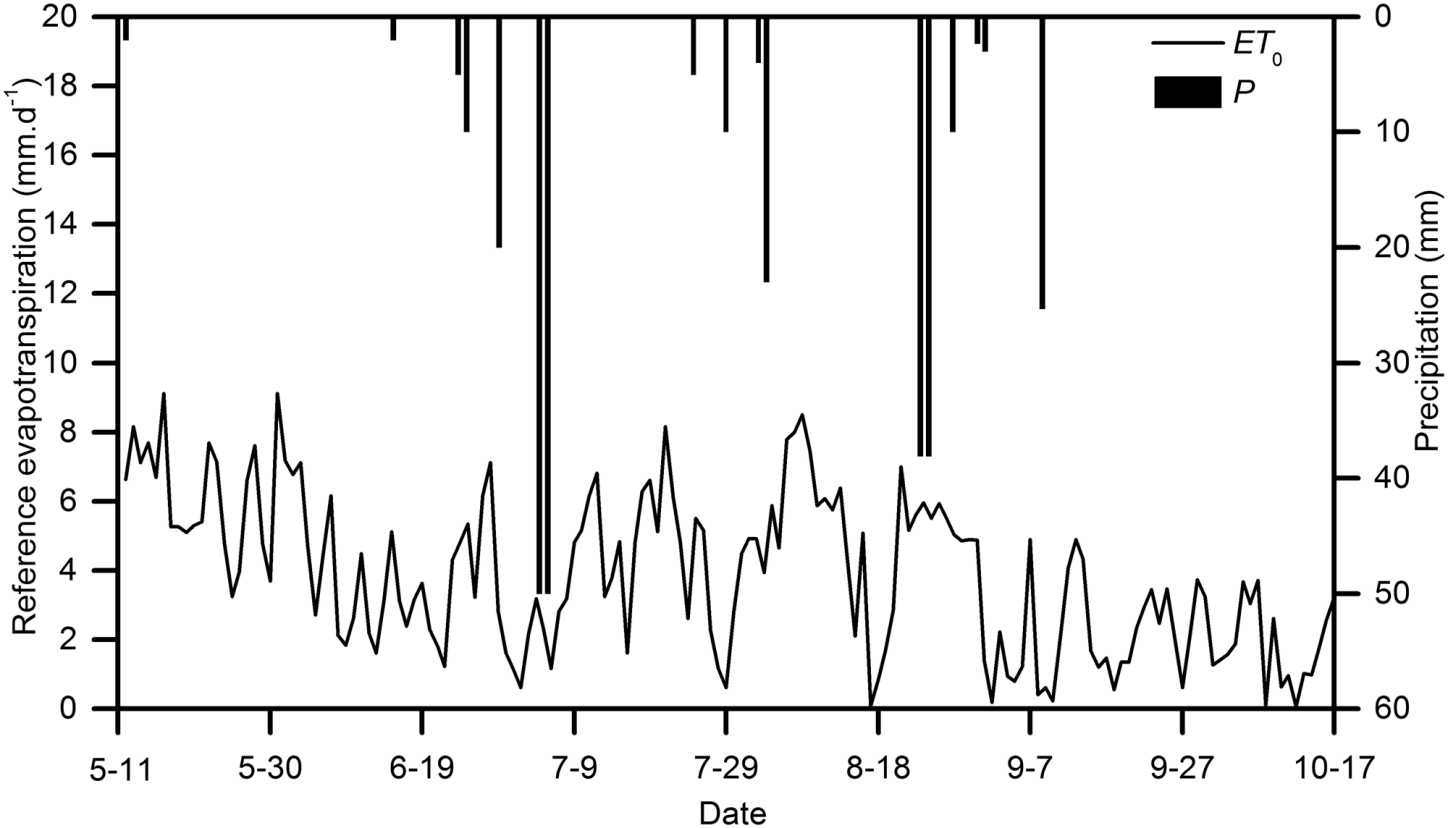
Diurnal variation of daily reference evapotranspiration rate and rainfall distribution in the whole growing season (1^st^ May–15^th^ October 2011).

The diurnal reference evapotranspiration (*ET*_0_) was calculated using the FAO 56 Penman-Monteith equation [13] on the basis of the *R*_n_, *T*_a_, *W*_s_, and RH measured by an automatic weather station over the experimental period as shown in Fig 2. The FAO 56 Penman-Monteith equation is as follows: *ET*_0_ equation:

$$ET_{0}=\frac{0.408\Delta (R_{n}-G)+ϒ\frac{900}{T_{\text{a}}+273}\mu_{2}(e_{s}-e_{a})}{\Delta +ϒ(1+0.34)\mu_{2}}$$ (1)

Where: *ET*_0_ is reference evapotranspiration (mm.d^-1^); *R*_n_ is Net radiation (W.m^-2^); *G* = SoilG is Soil heat flux density (W.m^-2^); *T*_a_ is average temperature (°C); *μ*_2_ = *W*_s_ is wind speed at 2 m height (m.s^-1^), *e*_*s*_ is saturated vapour pressure (KPa); *e*_a_ is actual vapour pressure (KPa); △ is vapour pressure slope of curves (KPa.°C^-1^); γ is psychrometric constant (65.5 KPa.°C^-1^).

### Leaf area index and leaf gas exchange measurements

A simple measurement of the amount of foliage by harvesting leaves (DOY 131, 150, 170, 178, 190, 210, 215, 230, 243, 250, 274, and 290) was performed. LAI (m^2^.m^-2^) was determined directly by taking a statistically significant sample of foliage from a plant canopy, measuring the leaf area per sample plot and dividing it by the plot land surface area.

Additionally leaf transpiration of the shrub with stem diameter of 25 mm within the experimental plot was measured with a gas exchange system (LI-6400; Li-cor, USA). The measurements were taken on an hourly basis from 18:00 to 08:00 (GMT +08:00) on 5^th^~ 6^th^ July, 2011 (DOY 186–187). The purpose of this measurement was to confirm if there was obvious nighttime transpiration in *H*. *scoparium*.

### Statistical analysis

We analyzed the correlations between sap flow and meteorological variables by using version 21.0 of the SPSS software (IBM Inc. NC, USA). Software Canoco for Windows 4.5 was used for redundancy analysis (RDA) to explore the responses of sap flow and environmental factors. The Figs were drawn using CanoDraw (Canoco 4.5; University of South Bohemia, Ceske Budejovice, Czech Republic). Data processing and plotting were completed with software OriginPro 9.0 (OriginLab Inc., Northampton, MA, USA).

Sap flow, physiology characteristics and environmental variables data from the present study are presented in S1 Dataset.

## Results

### Environmental characteristics of the study period

During the study period from May to October (DOY 131–290), 2011, the frost-free period was about 128 days.

**Fig 3:**
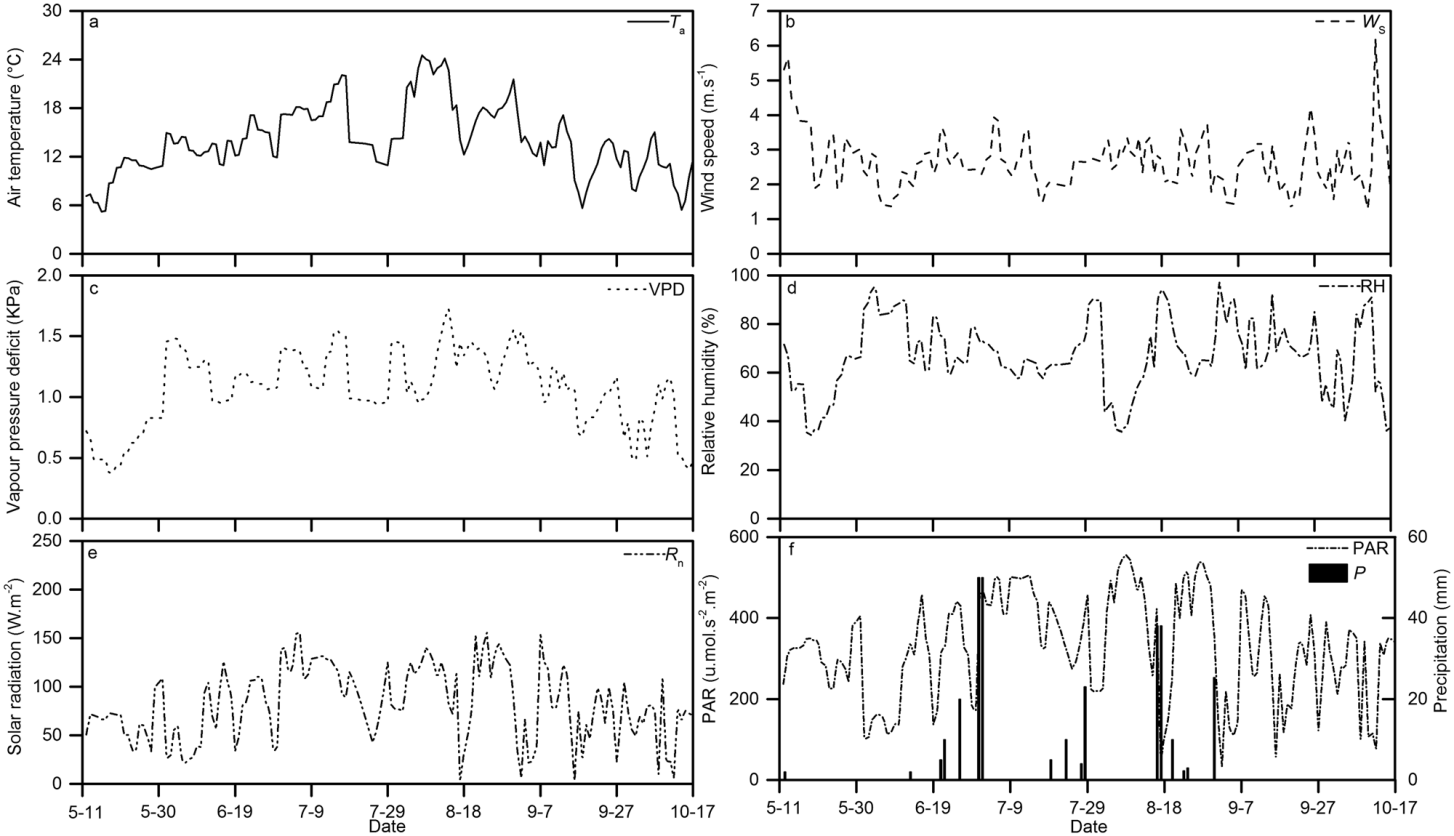
The patterns of variation in the meteorological variables during the measurement period.

Figs 2 and 3 illustrate the variations in the meteorological variables at the study site. Fig 2 exhibits that total *ET*_0_ during the study period was 938.57 mm, while P ranged from 1.20 to 50.00 mm per event with averaged 17.52 mm per event. In general P was highest during the summer in the form of intensive storm with short period typically less than a day. *T*_a_, VPD, *R*_n_ and PAR are shown in Fig 3 with the minimum values occurring mostly after a rainfall event followed by increasing values and reaching the maximum values before the next rainfall during the summer time. On the contrary, the RH values were positively correlated with precipitation densities. Throughout the study, average values of *T*_a_, VPD, RH, *R*_n_ and PAR were 13.95°C, 1.06 KPa, 66.63%, 81.48 W.m^-2^.day^-1^, and 323.90 μ.mol.s^-2^.m^-2^, respectively. In the study area, strong wind (>11 m.s^-1^) usually occurs in spring and winter. While during the study period, *W*_s_ values changed with time but extend of their changes was small, and the *W*_s_ averaged 2.42 m.s^-1^, with maximum and minimum values of 1.27 m.s^-1^ and 6.18 m.s^-1^, respectively.

**Fig 4:**
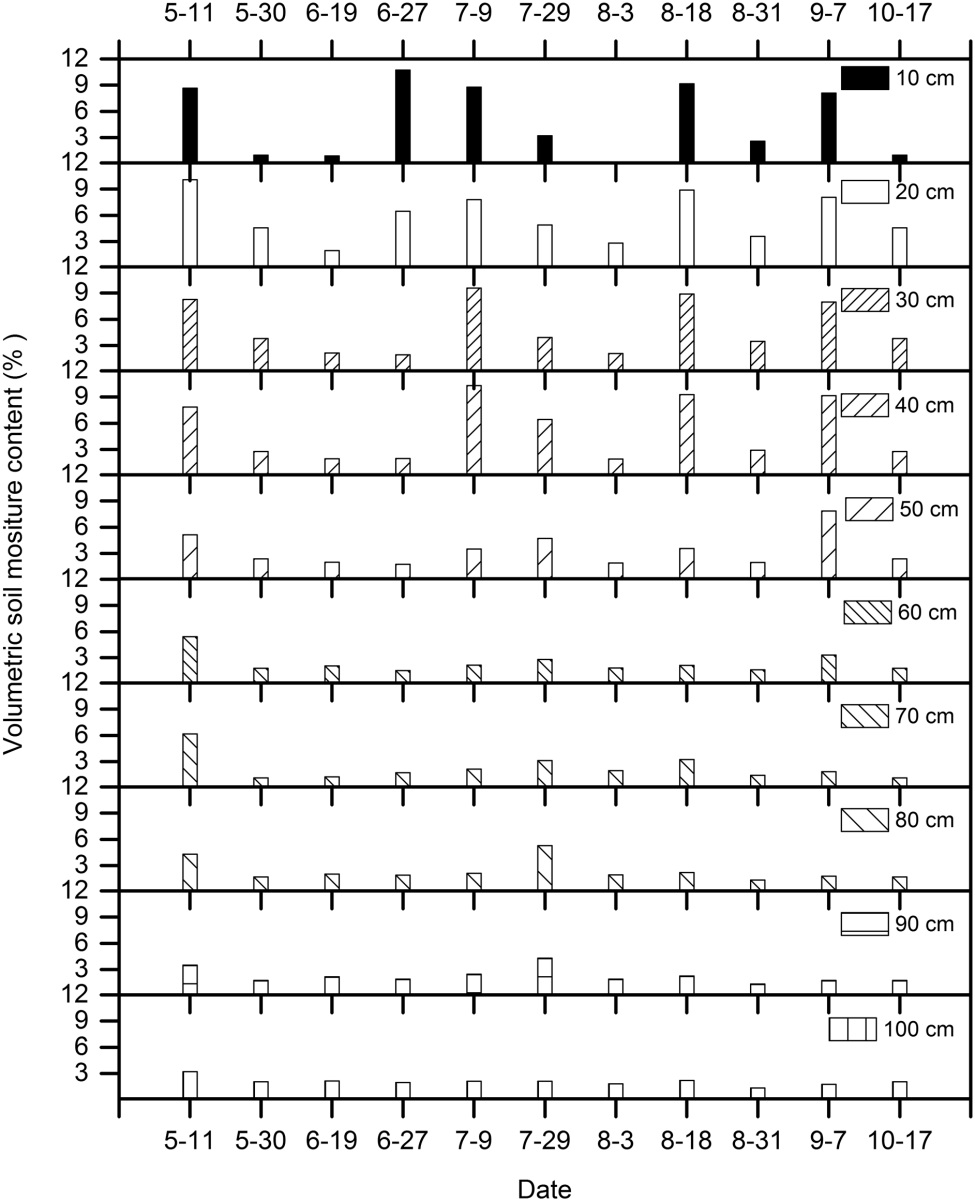
The seasonal variation of soil moisture contents measured in 0–100 cm soil layer during the measurement period.

**Fig 5:**
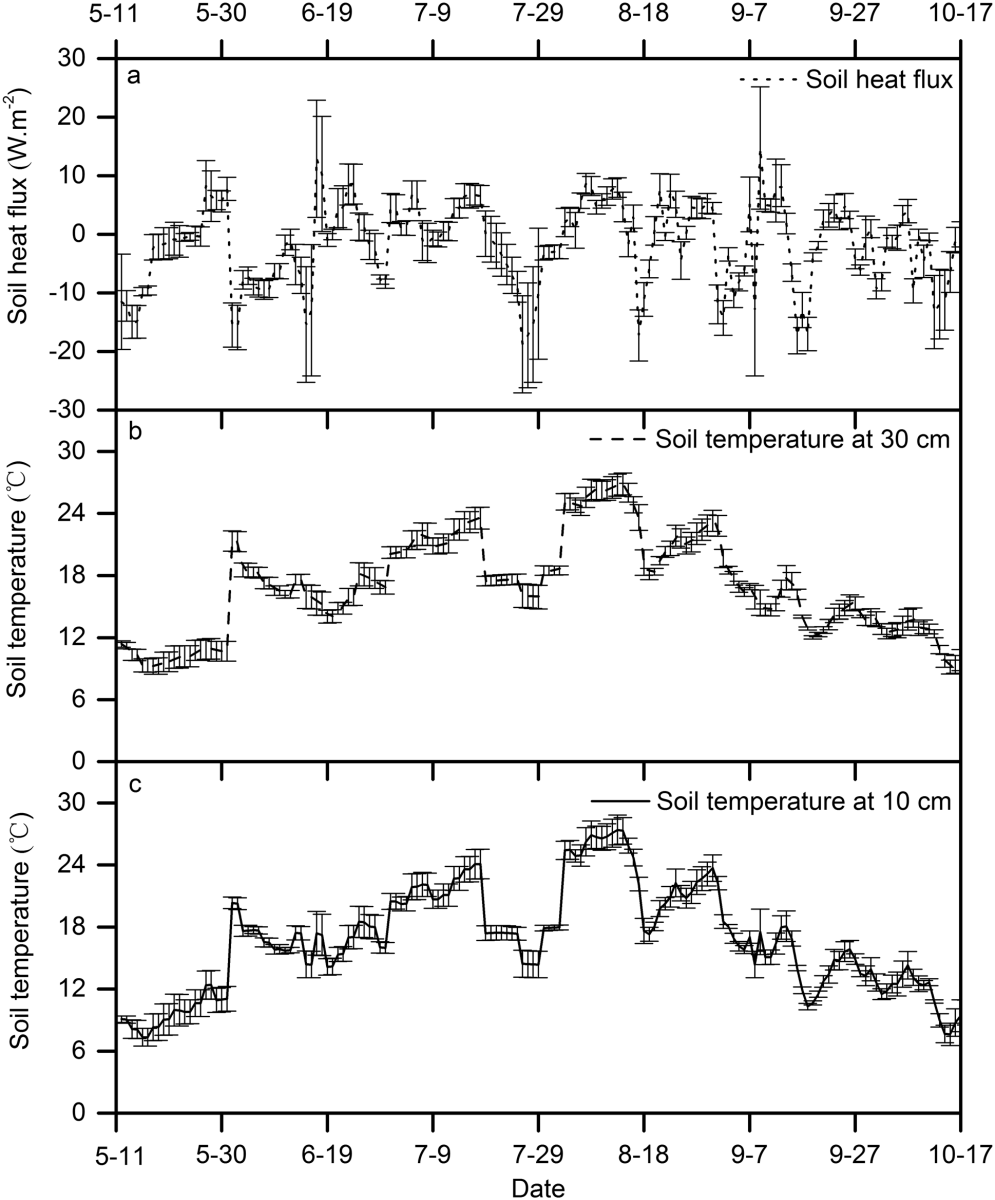
Seasonal variation of soil heat flux and soil temperatures during the measurement period.

SWC was higher after precipitation events, and particularly after large events. It generally ranged from 1.00 to 10.74% at soil depth of 0–100 cm. SWC was higher at a depth of 0–50 cm than at 60–100 cm and fluctuated markedly. It differed among months, with greater soil moisture in May, June, July, and August than in September and October (Fig 4). The SoilG is important in micrometeorology because it effectively couples energy transfer processes at the surface with energy transfer processes in the soil. The value and direction of the heat flux varied among the seasons due to the uneven rainfall distribution and intensive evaporation during the research period. During summer (June, July and early August), positive values of the SoilG (from atmosphere to soil) were observed as a result of the relatively unstable values of evapotranspiration and strong *R*_n_ (see Figs 2 and 3), in addition moist soil absorbs more heat than does loose, dry soil in the wet season. However, negative SoilG values were obtained in the spring and fall indicating that the heat transfer process was from the soil surface to the atmosphere (Fig 5a). Soil temperatures responded well to changes in the SoilG. The values differed among the seasons, and were higher in June, July and August than in September and October (Fig 5b and 5c).

### Diurnal and seasonal sap flow patterns

#### Diurnal variations of sap flow rate

The *J*_s_ in the stems of *H*. *scoparium* varied greatly during the measurement period because of natural heterogeneity and in response to environmental parameters. The experimental measurements also generated uncertainties due to the small sample sizes. In our study, the *J*_s_ values among the four plants were significantly correlated with each other during the entire growing season (the relations of the plant with a stem diameter of 25 mm with the other plants (16, 13, and 9 mm) (*R* ^2^ = 0.675, 0.673, and 0.703)). As shown in S3 Fig, the average values of *J*_s_ had a high dependence on stem diameter (standard deviations were in a reasonable range).

Thus to illustrate the changing diurnal trend, two consecutive clear days in each month were selected (DOY 132 and 133, 153 and 154, 186 and 187, 213 and 214, 267 and 268, and 280 and 290).

**Fig 6:**
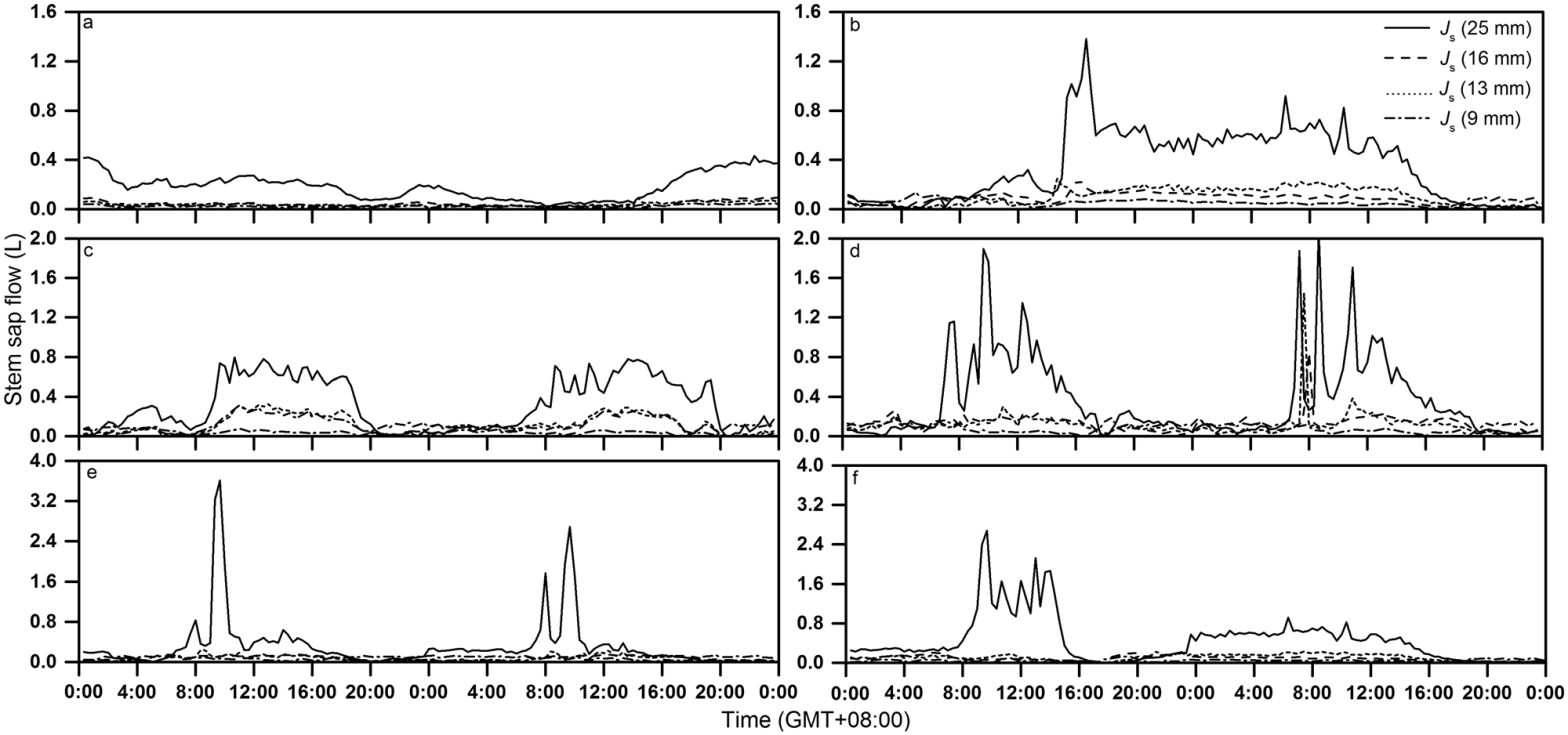
Dynamic variation in hourly sap flow rate, (a) May, (b) June, (c) July, (d) August, (e) September and (f) October in the whole growing season

Sap flow began at least 1 h before dawn and increased sharply during the first several hours of sunlight, increasing to midday (08:00–10:00 hours) as the *R*_n_ increased in intensity and the *T*_a_ increased. It reached a maximum between 10:00–12:00 hours (July, August, September and October), and then decreased after 16:00–17:00 hours (June)) before nightfall (Fig 6c, 6d, 6e and 6f).

As shown in Fig 6a and 6b, in May, there was substantial *J*_s_ at night and before dawn, indicating that the transpiration rate was low during the early stages of plant growth due to the small number leaves and physiological inactivity. As the plants grew, the *J*_s_ increased gradually after 08:00 hours, to a peak at 12:00 hours, and lasted for 13 h, with a value of 0.6 L.h^-1^ in June. Two factors contributed to this phenomenon. First, the vegetative shoots of *H*. *scoparium* continually produced leaves and lateral branches from late April. The photosynthetic rate of the leaves increased with the increase in leaf area, and the water uptake process developed from passive water absorption into transpiration pull. Second, a large amount of rainfall (about 135 mm) occurred in June. Soil water was saturated and provided the basic conditions for plants to grow, with a high transpiration rate meeting the growth needs during the day. The duration of water recharging at night was shortened, and during the night *J*_s_ almost stopped.

Unlike other desert shrubs [10, 12], *H*. *scoparium* had no significant ‘noon depression’. It is possible that the stomata of *H*. *scoparium* are not completely closed and transpiration therefore continues. Transpiration is influenced indirectly by leaf water potential through its effect on the stomatal aperture of plants. Reductions in transpiration have been attributed to decreases in leaf conductance in response to decreasing RH, thereby protecting leaf tissues from turgor loss and desiccation. In the semiarid areas, the increasing intensity of *R*_n_ and increasing *T*_a_ during the morning may induce stomatal opening, thereby accelerating *J*_s_ due to the high evaporative demand from the canopy. Therefore the primary response to the drought conditions is not to close the stomata.

However, the *J*_s_ pattern differed among months, and continued more substantially in spring and early summer than in autumn (compare Fig 6a and 6b to Fig 6e and 6f). The diurnal variation in sap flow rates was best described using an evident wide and multimodal curve in June, July and August, a bimodal curve in September.

#### Variations in the sap flow rate during the night

**Fig 7:**
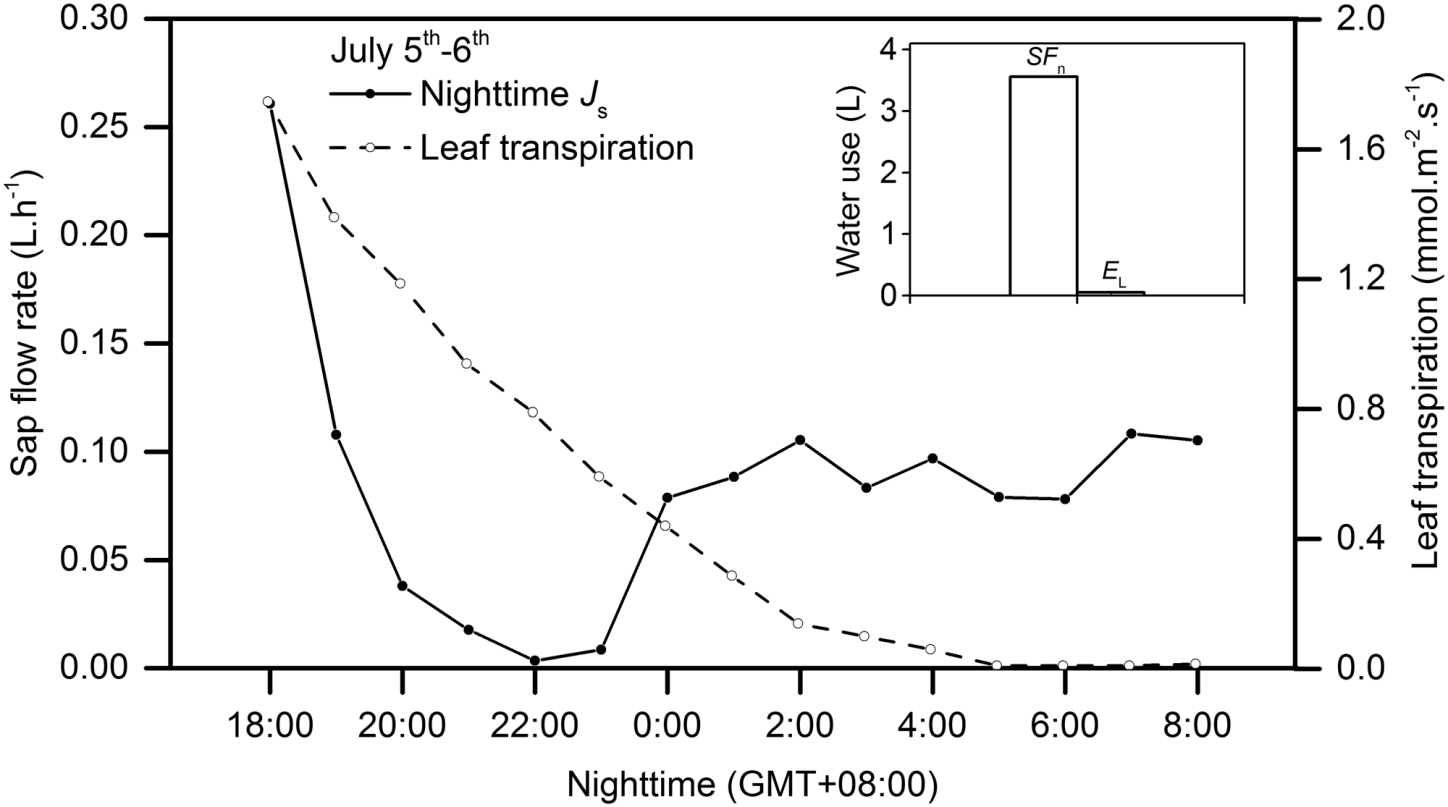
Dynamics of nighttime sap flow rate and leaf transpiration rate of *H*.*scoparium* (25 mm) during the nights of 5^th^–6^th^ July, 2011

In this paper, the nighttime range was determined between 18:00 to 06:00 hours when *R*_n_ was less than 5 W.m^-2^. It could also be a result of actual nighttime transpiration of the canopy or of recharging the depleted internal water storage of trees. In our studies, there existed substantial nighttime leaf transpiration in the studied shrub (Fig 7) that decreased sharply after 18:00 hours, slightly increased around midnight (0:00 hours and 02:00 hours), and then remained steady after 02:00 hours. We made a comparison between temporal dynamics of nighttime *J*_s_ and possible nighttime transpiration rates of canopy leaves in July 2011 to provide a basis for estimating the amount of stored water. During the most nighttime, the nighttime *J*_s_ did not follow the changing trend of leaf transpiration. The total accumulated water loss by canopy leaves (*E*_L_) was only 1.67% of the total nighttime sap flow (*SF*_n_). The result showed that the leaf transpiration of canopy comprises inappreciable fraction of the nighttime *J*_s_. The nighttime *J*_s_ is confirmed to be primarily used to recharge depleted internal water storages of trees.

As shown in the graph above (Fig 6), nighttime *J*_s_ fluctuated more significantly and was relatively higher before midnight, when it approached a steady state. The *J*_s_ after midnight was slightly higher in May, July, August, and September compared to that in June and October, which is consistent with the higher stem water recharge demand in summer. The nighttime *J*_s_ followed a seasonal trend similar to that in the daytime.

Our calculation showed that the contribution of daytime stem water recharge to the total transpiration of *H*. *scoparium* ranged from 51.53 to 80.41%, depending on the stem diameter and was considerably higher in the larger plants than the smaller plants. The contribution of nighttime stem water recharge to total transpiration was higher in plants with a smaller stem diameter. Over the whole growing season, the amount of nighttime *J*_s_ was considerable, reaching 19.59% (25 mm), 28.25% (16 mm), 21.76% (13 mm), and 48.47% (9 mm) of total daily *J*_s_.

#### Seasonal variations of sap flow rate

**Fig 8:**
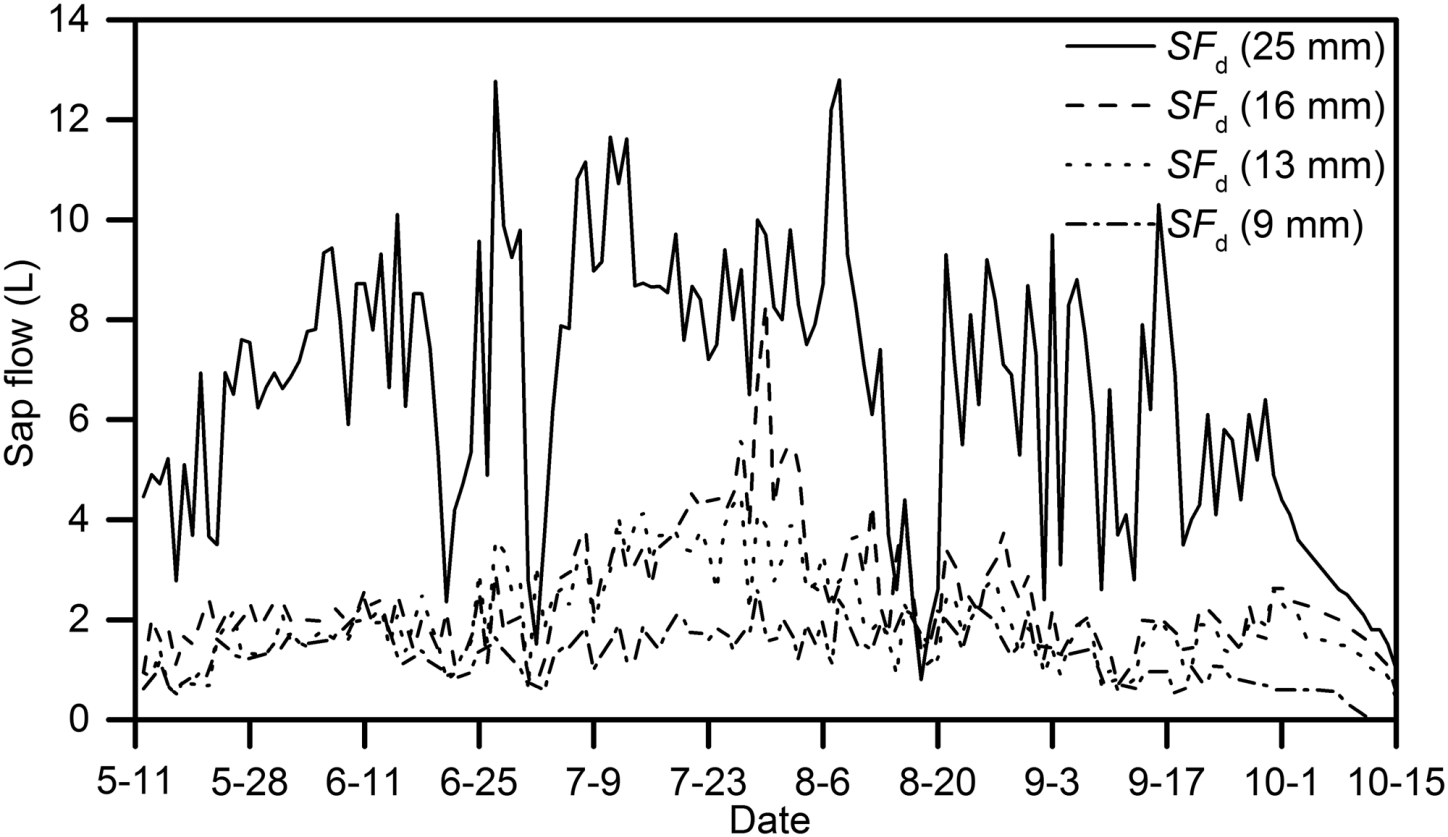
The seasonal variation of sap flow rate of *H*.*scoparium* (1^st^ May–15^th^ October 2011)

The *J*_s_ displayed different seasonal patterns. The *J*_s_ increased gradually from May and decreased gradually after August to a low and relatively steady value in October. The cumulative *J*_s_ of *H*. *scoparium* from June to August increased progressively due to the high level of water consumption during the long flowing period, high evaporation and abundant rainfall. Plants were later shed their leaves during the course of the experiment (Fig 8 and S4 Fig).

The cumulative *J*_s_ of the four plants (25, 16, 13, and 9 mm stem diameters) from May to August contributed the majority of the sap flow rate, which accounted for approximately 77–80% of the total *J*_s_. The minimum monthly cumulative *J*_s_ occurred in October, whereas the maximum was in June (25 mm) or July (16, 13, and 9 mm). The total water transpiration values of the four plants in the growing season (25, 16, 13, and 9 mm) were 1045.4, 397.7, 311.1, and 159.7 L, with maximum and minimum sap flow rates of 12.80 (8^th^ August) and 0.83 (18^th^ August), 11.76 (29^th^ July) and 0.95 (11^th^ May), 7.40 (29^th^ July) and 0.77 (22^th^ May), 3.54 (30^th^ July) and 0.62 L.d^-1^ (11^th^ May), respectively. The average water transpiration values per leaf area of the four plants during the growing season (25, 16, 13, and 9 mm) were 0.94, 0.92, 1.06, and 1.03 L.m^-2^.d^-1^, respectively.

Overall, the difference in *J*_s_ resulted mainly from the plant growth status. Among the four plants, *J*_s_ in the plants with a 25 mm stem diameter had the highest flow rates, with substantial fluctuations due to its physiological traits.

### The dependence of sap flow on meteorological variables

#### Correlation between sap flow, reference evapotranspiration and soil moisture content

**Fig 9:**
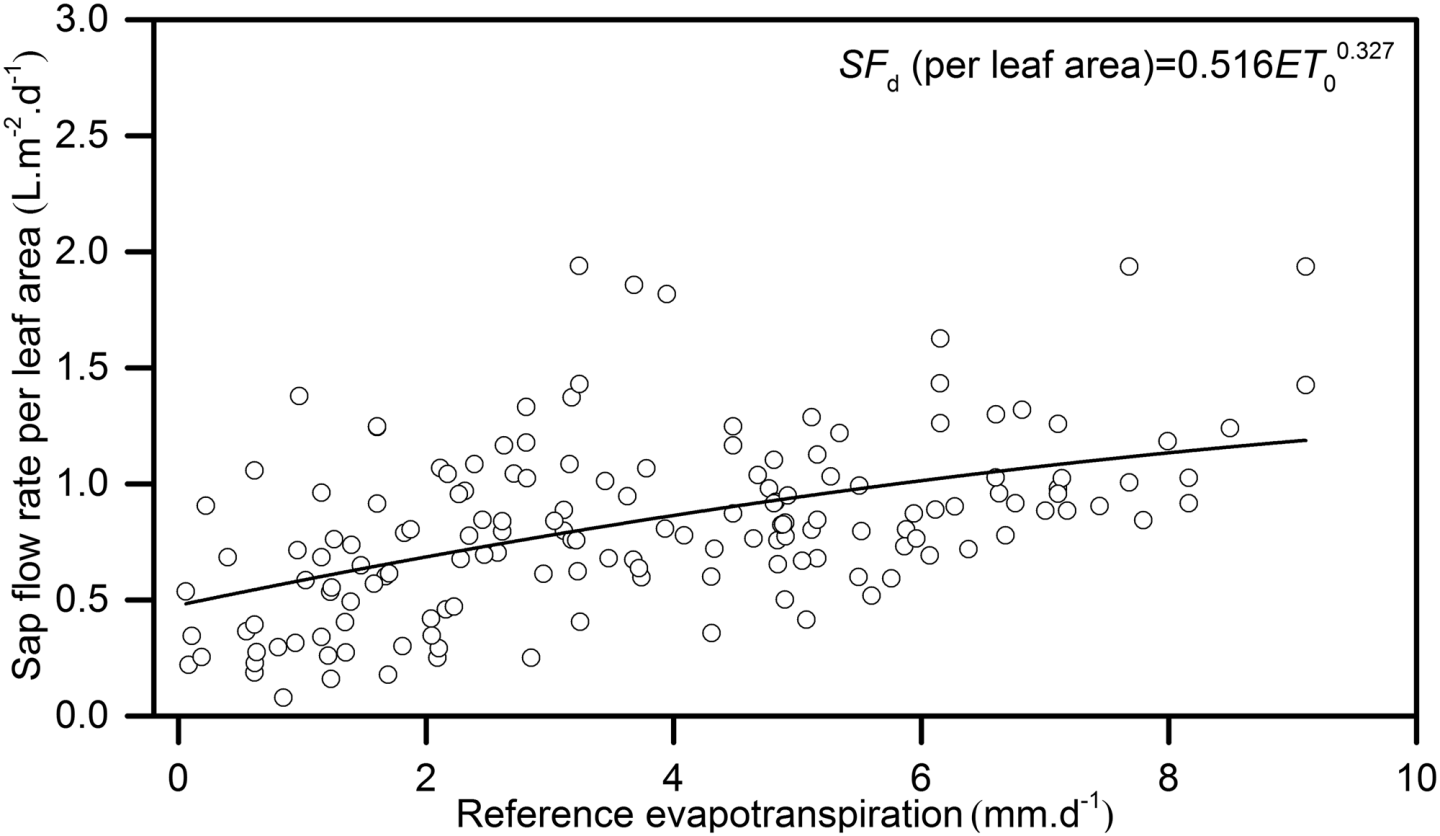
The relationship between daily sap flow rate per leaf area of *H*.*scoparium* and daily reference evapotranspiration rate.

The diurnal variation of *ET*_0_, was calculated by the Penman-Monteith model. At the daily time scale, there was a close relationship between the sap flow rate per leaf area and daily *ET*_0_ (Fig 9).

**Fig 10:**
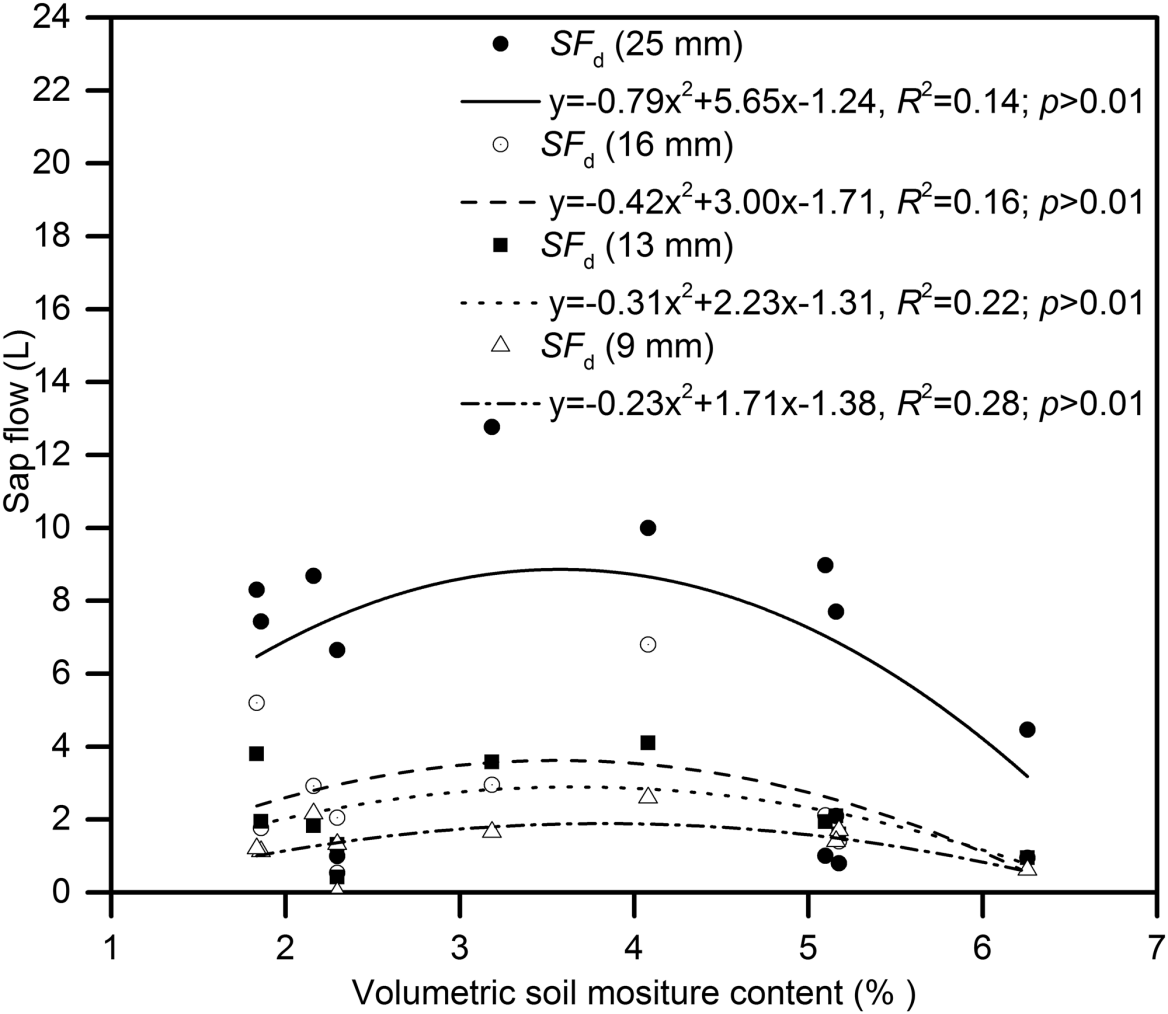
The relationship between daily sap flow rate of *H*.*scoparium* and soil moisture content.

The daily sap flow (abbreviated *SF*_d_ in Figs and Table) in *H*. *scoparium* was greatly affected by P, which can lead to SWC fluctuations causing daily changes in *J*_s_. However, because rain events were rare and the SWC rapidly declined after each rain event due to the high level of evaporation, the SWC was relatively low during the entire growing season, and had no direct relation with the daily *J*_s_ (Fig 10) Soil moisture levels increased during the rainy season (71% of annual precipitation occurs from June to August); however, *ET*_0_ was relatively high during these periods (especially in June). In contrast, in the early and late growing period, *ET*_0_ was relatively low, which led to a lower daily sap flow rate, indicating that the daily *J*_s_ was more closely coupled to changes in the *ET*_0_ (see Fig 9).

#### Variations of sap flow rate under different weather

**Fig 11:**
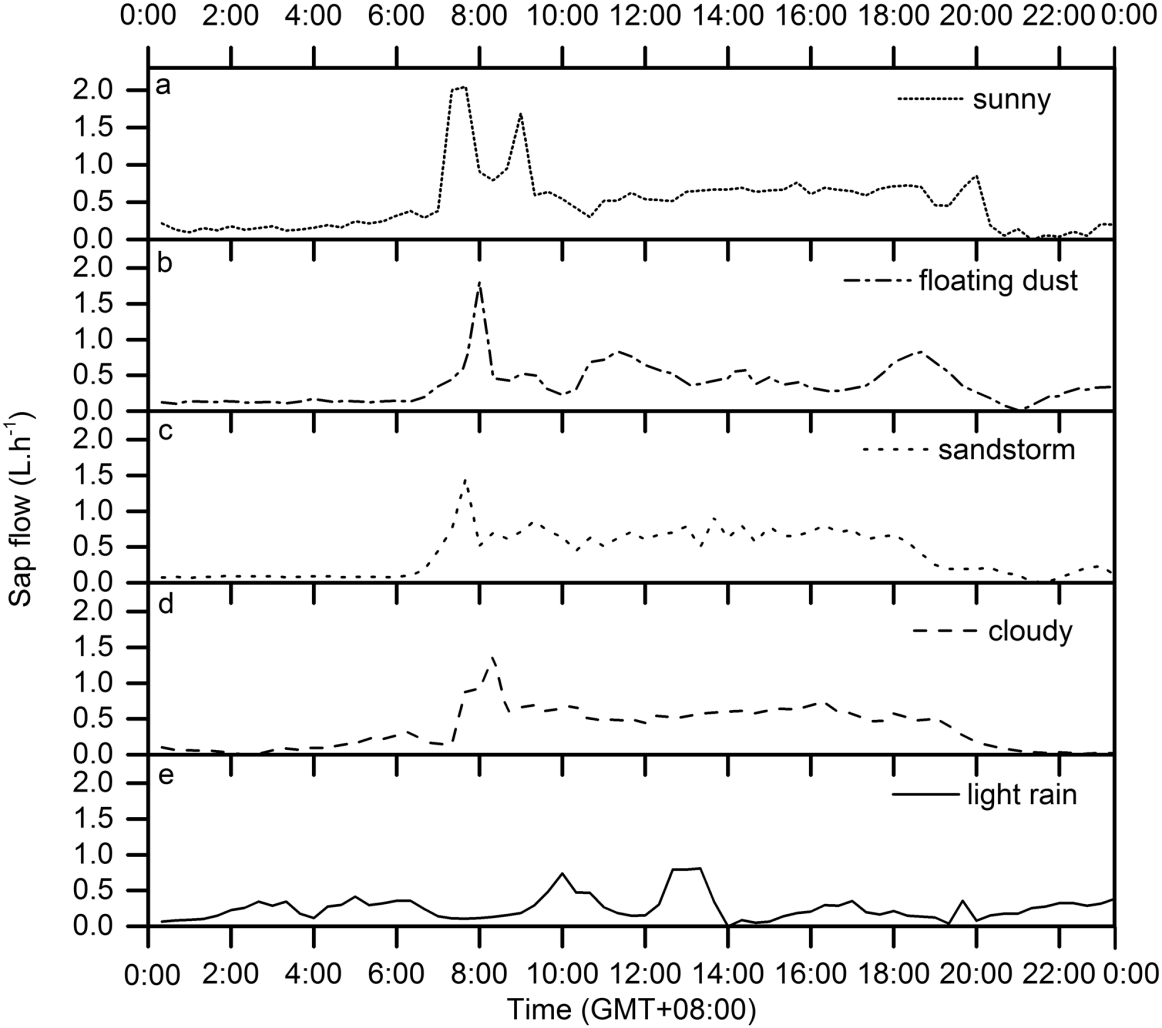
Fluctuation of hourly sap flow rate of *H*.*scoparium* (25 mm) under different weather conditions.

Environmental variables influence *J*_s_ through their effects on the plants’ physiological characteristics. In the whole growing season, the hourly *J*_s_ in the stem diameter of 25 mm plant was compared under typical weather days, e. g. sunny, flouting dust, cloudy, sandstorm and light rain days (DOY were 180, 227, 192, 69, 164 and 209, respectively). Fig 11 indicated that meteorological factors were the primary factors affecting the plants’ transpiration. The *J*_s_ reached the maximum in the sunny day of DOY 180, but the minimum in the rainy day of DOY 209. The *J*_s_ in the cloudy day were similar to those in the dust days. In sandstorm and dust days, high *W*_s_ can typically reduce water loss and cause the stomatal closure [11]. Rainy weather suppressed *J*_s_ due to the increased RH, reduced VPD and *R*_n_. In contrast, high *J*_s_ was caused by high *R*_n_, *T*_a_, and VPD in sunny weather. The order of mean *J*_s_ under different weather conditions are: sunny > cloudy > sandstorm > flouting dust > light rain days.

#### The response of sap flow to environmental factors

**Fig 12:**
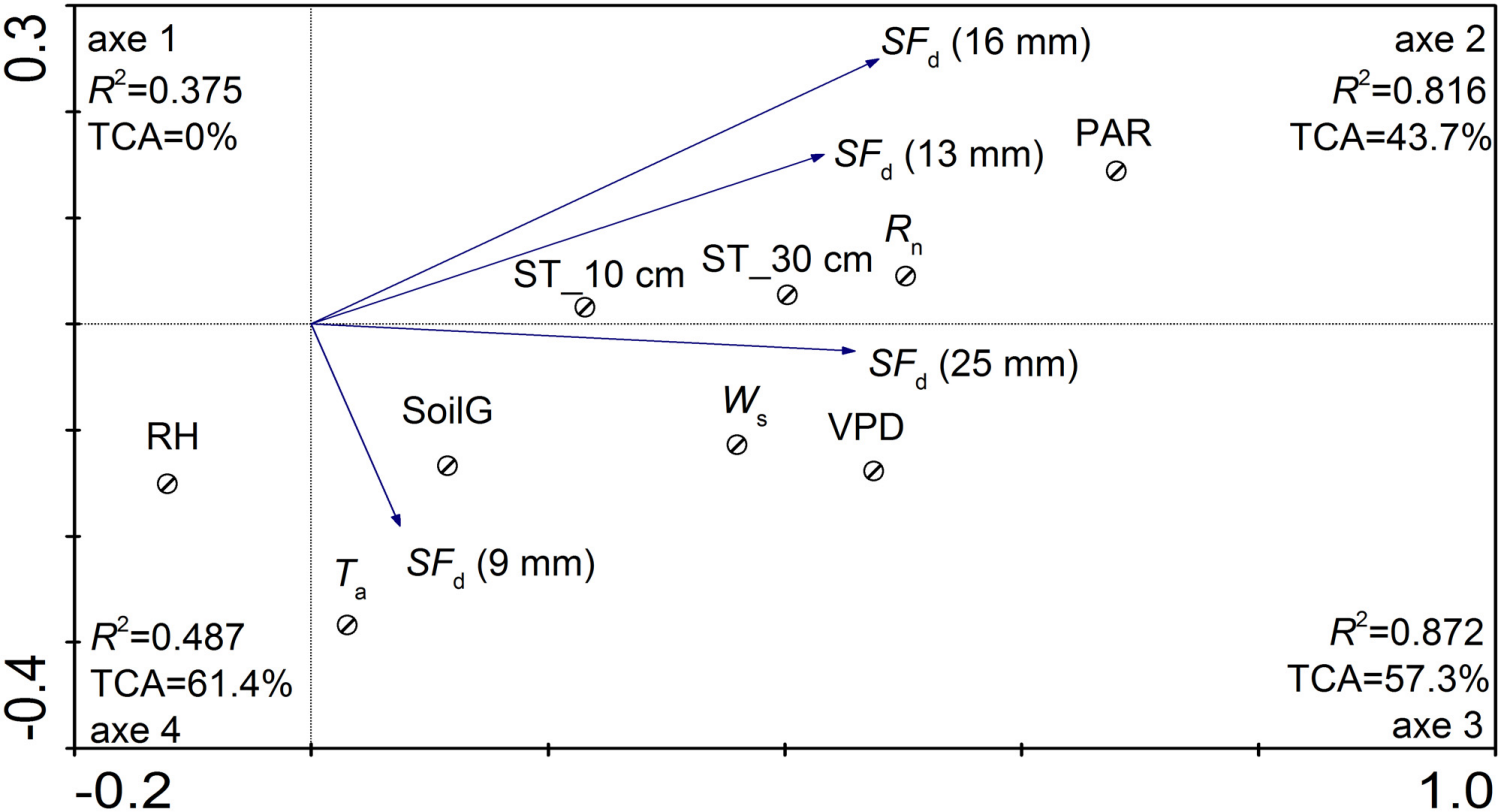
Redundancy analysis for the relationship between daily sap flow rate and the environment variables.

The daily *J*_s_ in *H*. *Scoparium* (25, 16, 13, and 9 mm stem diameters) varied in response to environmental conditions (Fig 12). In Fig 12, the second, third, and fourth axes can explain most of the variation. The three RDA graphs indicated a strong correlation between the *J*_s_ values of *H*. *Scoparium* and natural environmental conditions, suggesting that most of the environmental variables are significant for plant transpiration (Table 1). The correlation between the *J*_s_ and meteorological variables was high for the second and third canonical axes (*R* ^2^ = 0.812 and 0.876 respectively) and the total cumulative variance (TCA) accounting for two axes totaled 43.7 and 57.5%, which suggests that *J*_s_ values in *H*. *Scoparium* (25, 16, 13, and 9 mm) were strongly correlated with these axes.

**Table 1:** Kendall’s tau correlation matrix between sap flow and the meteorological variables^a^.

Diameter/Factors		*R*_n_	PAR	*T*_a_	VPD	*W*_s_	RH	ST_10 cm	ST_30 cm	SoilG
**25 mm**	Whole time	0.135	0.280**	0.0690	0.131	0.222**	-0.301**	0.0720	0.0860	0.0910
	Daytime	-	-	0.439**	0.463**	0.234**	-0.147	-	-	-
	Nighttime	-	-	0.0930	-0.004	-0.151	-0.0380	-	-	-
**16 mm**	Whole time	0.326**	0.349**	0.216**	0.265**	0.249**	-0.228**	0.254**	0.249**	0.247**
	Daytime	-	-	0.316**	0.580**	0.579**	-0.102	-	-	-
	Nighttime	-		0.0610	0.0390	-0.0210	-0.0200			
**13 mm**	Whole time	0.280**	0.332**	0.156*	0.248**	0.188*	-0.335**	0.182*	0.172*	0.176*
	Daytime	-	-	0.505**	0.650**	-0.480**	-0.116	-	-	-
	Nighttime	-	-	0.111	0.00800	-0.086	-0.0330	-	-	-
**9 mm**	Whole time	0.344**	0.188*	0.650**	0.587**	0.311**	-0.005	0.622**	0.648**	0.651**
	Daytime	-	-	0.203	0.230	-0.101	-0.173	-	-	-
	Nighttime	-	-	0.231*	0.256*	0.231*	-0.123	-	-	-

^a^*and**mean they are significant at *p*<0.05 and *p*<0.01, respectively

The RDA and Kendall’s tau values indicated that *R*_n_ and PAR had the strongest influence on daily *J*_s_ (25, 16, 13, and 9 mm), *R* ^2^ = 0.135, 0.326, 0.280, 0.344 and 0.280, 0.349, 0.332, 0.188, respectively. RH had the least effect on *J*_s_. The SoilG and ST were significantly positively correlated with *J*_s_ (*p* < 0.01), and *J*_s_ was more strongly affected by ST than by SoilG. In addition, VPD, *T*_a_ and *W*_s_ were significantly positively correlated with *J*_s_ (*p* < 0.01) (Table 1).

We expressed the variation in daily *J*_s_ in plants with a stem diameter of 25 mm by means of a stepwise linear regression against the meteorological factors. The resulting model performed well, explaining 70.0–89.0% of the variation in sap flow rates (Table 2). Taking into account the self-correlation of environmental factors, the optimal regression model indicated that the daily *J*_s_ was significantly correlated with *T*_a_, VPD, and soil conditions, with a strong, but insignificant, correlation with RH over the whole growing season, which was in agreement with the RDA analysis.

**Table 2:** The stepwise linear correlation equations between daily sap flow and environment factors in different growth stages.

Month	Regression equations	Intercept	*T*_a_	VPD	*W*_s_	RH	ST_30	SoilG	*R* ^2^	*F*	*n*
**May**	*SF*_d_ = *f* (RH)	3.60	-	-	-	-0.024	-	-	0.73	21.83**	20
**June**	*SF*_d_ = *f* (RH)	-0.39	-	-	-	0.87	-	-	0.76	7.18**	30
**July**	*SF*_d_ = *f* (RH)	12.38	-	-	-	-0.097	-	-	0.82	12.34**	31
**August**	*SF*_d_ = *f* (RH, *W*_s_)	46.65	-	-	10.60	-0.16	-	-	0.86	45.24**	31
**September**	*SF*_d_ = *f* (VPD)	-1.19	-	2.58	-	-	-	-	0.76	22.34**	30
**October**	*SF*_d_ = *f* (ST_30, SoilG)	28.97	-	-	-	-	-1.88	0.16	0.70	18.42**	20
**Whole season**	*SF*_d_ = *f* (*T*_a_, VPD, ST_30, SoilG)	-1.38	-0.099	0.76	-	-	0.19	0.024	0.89	133.89**	162

#### Daytime and nighttime sap flow driving force

**Fig 13:**
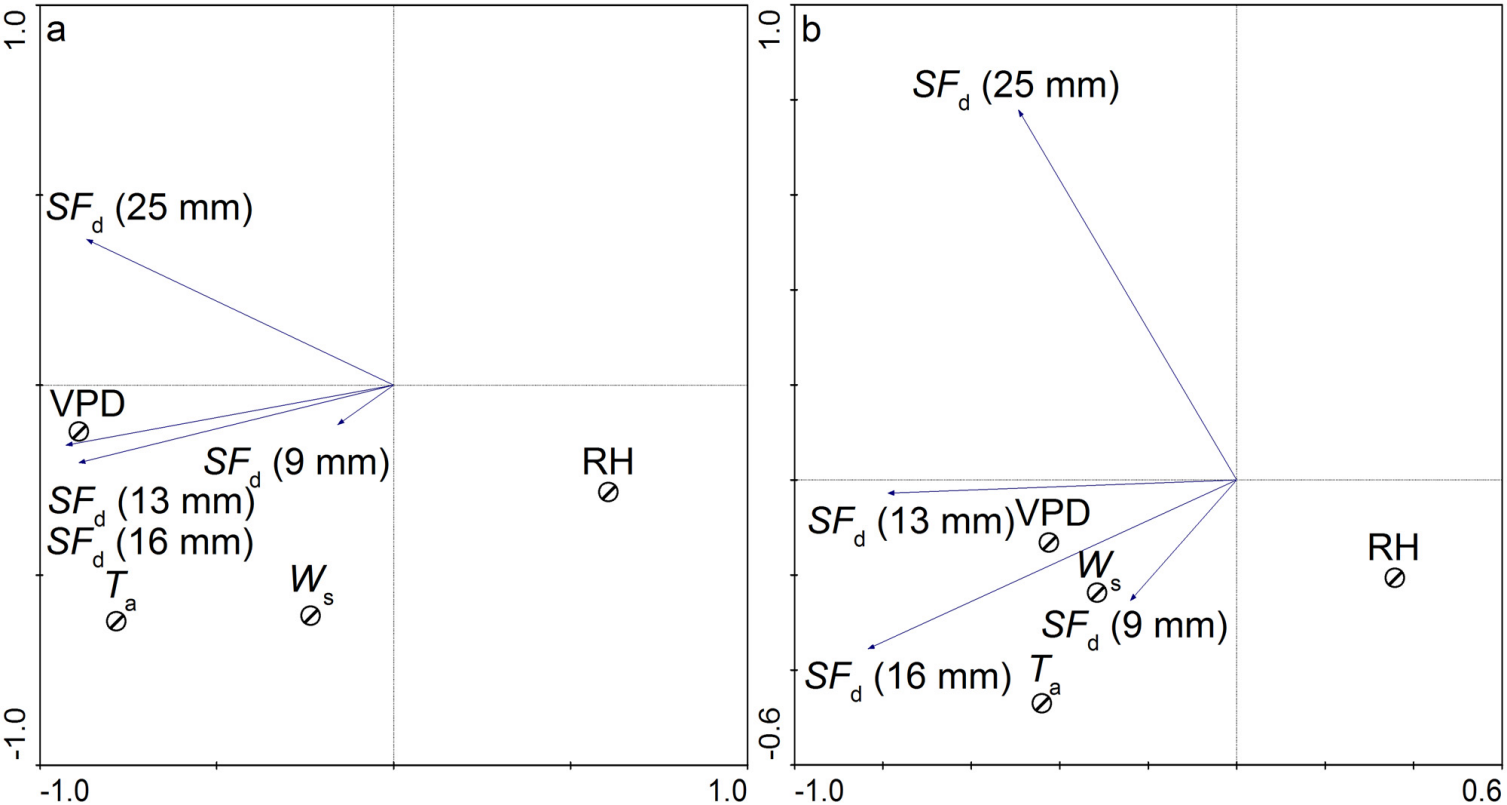
Redundancy analysis for the relationship between (a) daytime and (b) nighttime sap flow rate and the meteorological variables.

A comparison of the day and nighttime *J*_s_ of *H*.*scoparium* (25, 16, 13, and 9 mm stem diameters) in response to climate conditions is shown separately in Fig 13. Because radiation is absent during the nighttime, the interaction of environmental driving factors is different from that during the daytime. To eliminate the side effects of soil conditions (ST and SoilG), *J*_s_ data for rainy days were not applied and *R*_n_ and PAR factors were also excluded. Correlation analyses of both the daytime and nighttime *J*_s_ of *H*. *scoparium* and *T*_a_, VPD, *W*_s_, and RH in the growing season of 2011 were conducted.

The results showed that nighttime *J*_s_ had different meteorological driving patterns from daytime *J*_s_, and sap flow rates responded to meteorological factors differently depending on the stem diameter. According to the RDA analysis, the *J*_s_ of *H*. *scoparium* (25, 16, 13 and 9 mm) and environmental variables were more clustered in daytime than in nighttime (e.g., compare Fig 13a and 13b). While during the nighttime, there was a higher correlation between the *J*_s_ of *H*. *scoparium* and meteorological variables in the plants with a 9 mm than those of the 13, 16, and 25 mm diameter plants, suggesting that the nighttime transpiration in small plants was more sensitive to meteorological variables.

The RDA and Kendall’s tau values indicated that both daytime and nighttime *J*_s_ were significantly correlated with *T*_a_ and VPD, but were negatively correlated with *W*_s_ and RH (Table 1). The correlation between the *J*_s_ and meteorological variables was high for the fourth canonical axes and the TCA that accounted for this axe in daytime and nighttime totaled 95.8 and 93.4%, respectively (data not shown).

**Fig 14:**
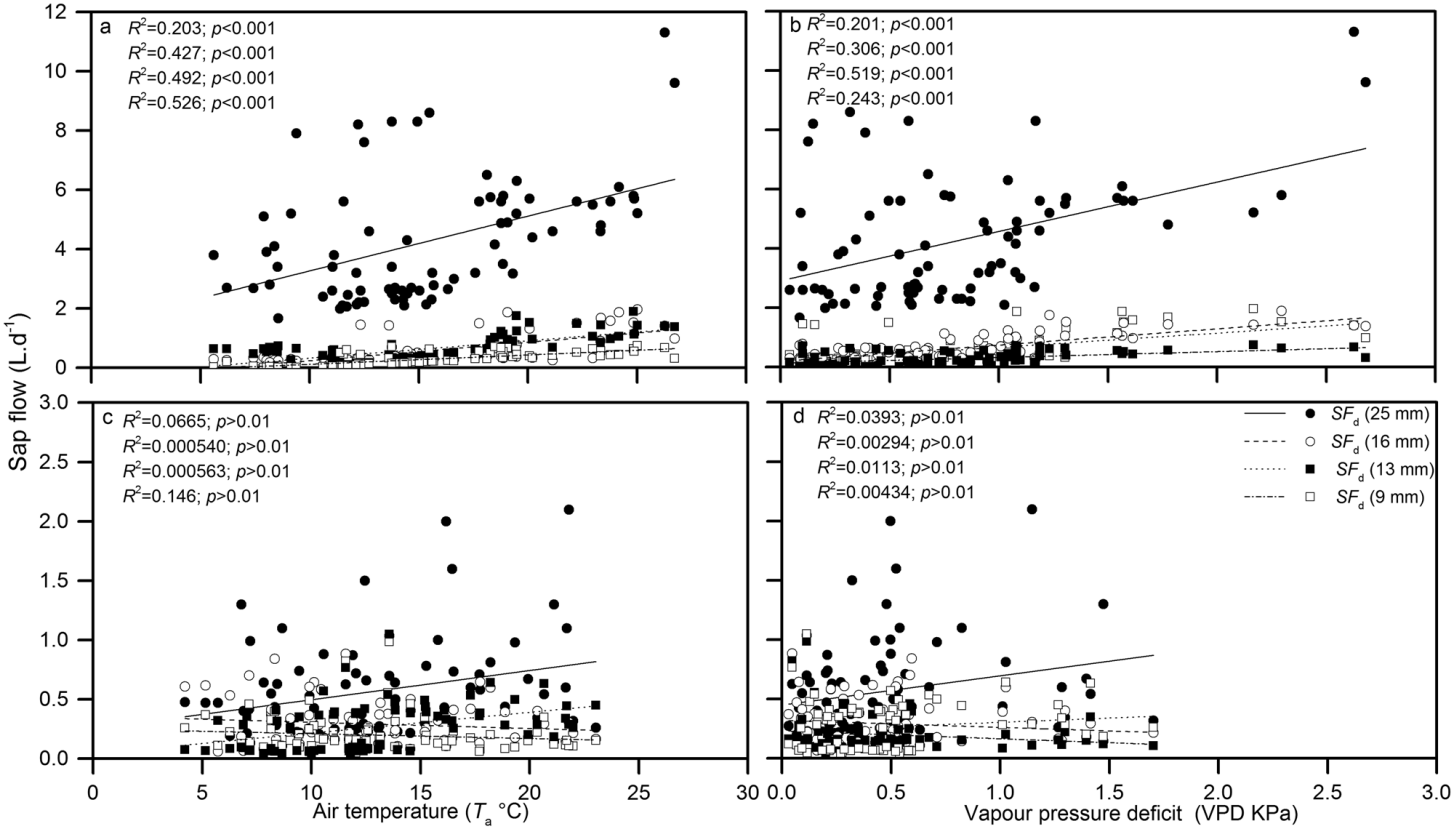
Sensitivity of daily sap flow rate to air temperature at (a) day and (c) night. Sensitivity of daily sap flow rate to vapour pressure deficit at (b) day and (d) night during the day in 2011.

Regression analysis showed that there was a significant correlation between *J*_s_ and *T*_a_ and VPD, but neither *T*_a_ nor VPD did not adequately explained the variation in the nighttime *J*_s_. The sensitivity of nighttime *J*_s_ to *T*_a_ and VPD differed significantly from the sensitivity of daytime *J*_s_ to *T*_a_ and VPD. The regressions describing the relationship of the two variables were significant in the daytime, but not the nighttime. However, the sensitivity of nighttime sap flow rates to *T*_a_ in the 25, 16, and 13 mm stem diameter plants were much lower than in the 9 mm stem diameter plants, while for VPD the highest *R* ^2^ in the daytime was for intermediate sized plants and in the nighttime for the 25 mm stem diameter plant (Fig 14).

**Fig 15:**
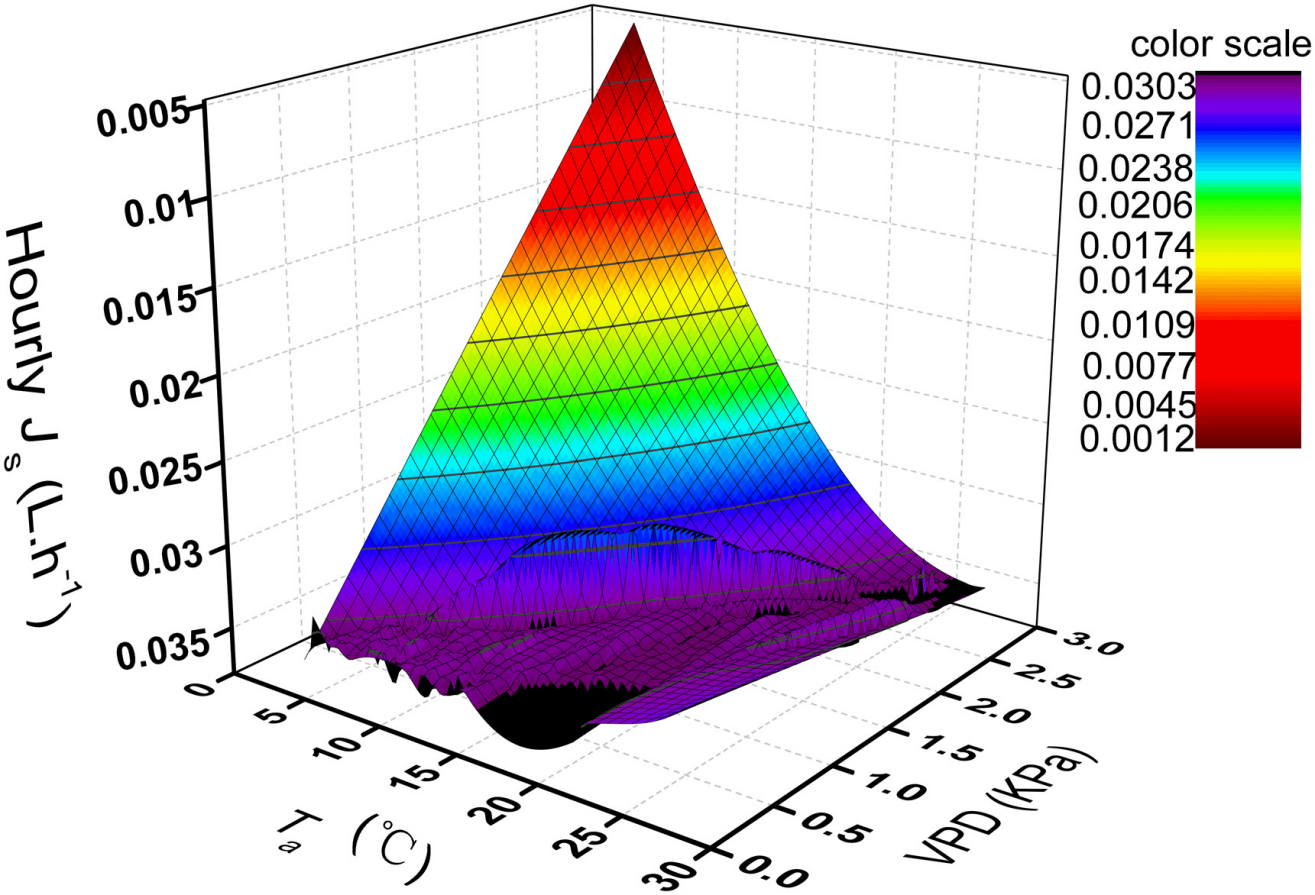
Hourly sap flow rate response to instantaneous values of vapour pressure deficit and air temperature for *H*.*scoparium* (9 mm) Z axis units are hourly integrated estimations.

In previous studies, a significant linear correlation between the nighttime *J*_s_ and both *T*_a_ and VPD was identified [14–17]. During our research, the interactions between the nighttime *J*_s_ of *H*. *scoparium* and climatic factors in this region were more complex. This may imply that the nighttime *J*_s_ of *H*. *scoparium* was influenced by the combined effect of both *T*_a_ and VPD. Thus, we analyzed the relationship between the hourly *J*_s_ (L.h^-1^) of *H*. *scoparium* (9 mm) and both *T*_a_ and VPD. The results revealed that high *T*_a_ and VPD values could lead to high plant transpiration. The *J*_s_ varied greatly when *T*_a_ and VPD increased. From Fig 15, the minimum threshold of VPD driving force can be seen to be around 1.5 KPa and the optimal *T*_a_ was about 20°C. The nighttime driving force was maximized when *T*_a_ was high and an elevated VPD was observed on many nights (usually from nightfall to midnight during summer) during the study.

## Discussion

Measurements of *J*_s_ have been widely used to study plant responses to environmental factors. In our experiment, the diurnal stem *J*_s_ of *H*. *scoparium* varied regularly and displayed consistent patterns of day and night cycles in plants with different stem diameters. In semi-arid regions, because of the large daily transpiration, tree roots passively absorb water, resulting in a large *J*_s_ with a bimodal or multimodal curve. This behavior is universal among similar desert plant species, such as *Haloxylon ammodendron* [18], *Salix gordejevii* [19], *Caragana microphylla* [15], *Nitraria sphaerocarpa* and *Elaeagnus angustifolia* [10]. During nighttime, *J*_s_ still occurs and becomes constant to alleviate water stress and achieve a water balance in the plant body. In this study, the nocturnal *J*_s_ was very stable than the daytime *J*_s_ and reveals that there were minor turbulence changes in nighttime environmental variables with the *J*_s_ responding to atmospheric drivers [20–23].

It is commonly assumed that transpiration does not occur at night because leaf stomata are closed in the absence of light [17]. However, there is considerable evidence that the stomata of some species do not close completely during the night which allows for transpiration in the presence of sufficient environmental driving forces [24]. Our study revealed that the nighttime *J*_s_ of *H*. *scoparium* was substantial and varied according to the time of year and at different times of the night. Nighttime *J*_s_ was correlated with the daytime *J*_s_ of the previous day. This is consistent with the results of Snyder’s study which revealed that higher nighttime stomatal conductance and transpiration associated with higher daytime values across species and habitats, implying that quite an amount of nighttime *J*_s_ was used to refill the stem water deficit as a result of high water loss during previous daytime [25].

Leaf gas exchange and stem heat balance measurements were conducted in order to investigate the partition of nighttime *J*_s_. It was found that the *E*_L_ was only 1.67% of the *SF*_n_ (see Fig 7). Therefore, it is likely that the nighttime *J*_s_ we observed was mainly used for refilling water in the trunk. Same to our results, nocturnal *J*_s_ was found to be primarily a function of refilling of stem storage rather than transpiration from the canopy with canopy transpiration accounting for 2.6–8.5% of nocturnal flows in *Acacia mangium* in the hilly lands of subtropical South China [26].

Plant size is often the major parameter determining the water storage capacity [26]. During our studies, the nighttime water recharge of *H*. *scoparium* was strongly dependent on stem diameter. Our estimates of the contribution of nighttime water recharge to the total transpiration ranged from 19.59 to 48.7% depending on the stem diameter of the plant. The contribution of nighttime stem water recharge in *H*. *scoparium* was higher in summer than other seasons.

During the entire experimental period, the total sap flow rates of *H*. *scoparium* were 1045.4 (25 mm), 397.7 (16 mm), 311.1 (13 mm) and 159.7 L (9 mm). Sap flow rates increased from May to August, and then decreased from September to October due to changes in the local natural environment. There was a significant exponential relationship between *J*_s_ and the *ET*_0_ on a daily time scale. On an hourly time scale, the relationship was influenced by P [27]. Usually, a small amount of P would increase the *J*_s_ and the *ET*_0_, but when the P was large, the *J*_s_ and the *ET*_0_ decreased sharply. The reason for this was that a small amount of rainfall would increase the leaf water potential and leaf conductance to the water VPD, gas exchange and photosynthesis, but when the rainfall was large, the *R*_n_, *T*_a_, and VPD would decline over a long period (see Figs 2 and 3), and hence the *J*_s_ and *ET*_0_ decreased [27]. In the experimental period, 25 individual rainfall events were observed, with over 70% occurring in summer. The rainfall occurred as strong and short-term events, and afterwards long-term drought stress occurred on the soil surface. Thus, in our research, *J*_s_ data during rainy days was not used in the correlation analysis because the duration and amount of P were crucial for determining the *J*_s_ values, which was dependent on the resulting variations in meteorological factors, SWC and soil water limitations can cause a decreased *J*_s_ and high evaporative demand may result in data errors and inaccuracy [11].

SWC in the 0–100 cm layer, with P mainly accumulated at this infiltration depth. In the previous studies, the relationship between the SWC and *J*_s_ was shown to be complex. Xia et al. (2008) reported that SWC had no direct relationship with the daily *J*_s_ [11], but contrary results have also been reported [27–29]. In our study, no obvious evidence of strong close correlations between *J*_s_ and the shallow SWC levels (0–100 cm) was found, but the close relationship between *J*_s_ and the *ET*_0_, indicated that the transpiration rate of *H*. *scoparium* was more responsive to the changes of *ET*_0_. In northwestern China, regional climate change is likely to increase the variability in P patterns. Consequently, desert shrubs will be forced to endure repetitive cycles of water scarcity followed by uneven rainfall. As a high water consumption plant, in *H*. *scoparium*, natural rainfall in the shallow soil layer is not fully utilized. It is therefore possible that *H*. *scoparium* may consume underground water (in the region the typical underground water depth is below 15 m) to maintain growth and drought resistance. In some locations, *H*. *scoparium* may have a negative impact on the water equilibrium due to its deep root system and potential water consuming capacity, which requires further investigation.

The variation of *J*_s_ in tree species was related not only to their biological and physiological characteristics, such as canopy structure, stomatal closure and root hydraulic conductance, but also to environmental factors [9, 27, 28]. In the whole growing season due to rapidly changing weather, there were many outliers data points in the *J*_s_ data values which were very sporadic, jumpy and inconsistent, most of which were observed in the 25 mm stem diameter plant. Indicate that larger plant was more sensitive to climatic factors. Because of its broad transpiration leaf area, the stem diameter of 25 mm plant had the largest flow rate and a great fluctuating variation.

In our study, *J*_s_ varied under different weather, agreeing with a Giorio and Giorio (2003) who reported that the magnitude of *J*_s_ for olive trees during sunny days was greater than during rainy days due to low *T*_a_, low *R*_n_ and a low VPD [30]. Similar trends were also found in *Larix decidua* [31], *Eucalyptus grandis* [32, 33], *Ligustrum japonicum* [34] and *Tamarix elongata* [28] that low *J*_s_ can be caused by low VPD in windy weather; increasing intensity of *R*_n_ and increasing *T*_a_ on sunny days induce stomatal opening in a certain time, thereby accelerating *J*_s_.

RDA and Kendall’s tau values suggested that daily *J*_s_ of plants was correlated positively with *R*_n_, *T*_a_, and *W*_s_, and negatively with RH (*p* < 0.01). The daily *J*_s_ (25 mm) in *H*. *scoparium* was found to be a function of six environmental factors (*T*_a_, VPD, *W*_s_, RH, soil temperature at 30 cm, and SoilG) but all varied throughout the year. The same methods were applied in *Juglans regia* L [28], *Malus domestica Borkh* [35], *Caragana korshinskii* [11], *Caragana microphylla* [12] and *Populus euphratica* [24], and it was found that *T*_a_, VPD, and RH were the three major factors affecting sap flow rate on clear days, but the role of the three factors varied according to the growth stages.

It has previously been suggested that environmental factors, such as *T*_a_ and VPD, significantly affect nighttime *J*_s_ [22, 24, 26, 33, 36–41]. The significant correlation between some environmental factors and the nighttime *J*_s_ of *H*. *scoparium* observed in this study supports this assertion (see Table 1). In addition, the nighttime *J*_s_ was more affected by tree features, especially in the plants with a smaller diameter. The nocturnal *J*_s_ of small plants corresponded to *T*_a_ and VPD at the study site, which can be approximated by a non-linear relationship based on the minimum threshold for nighttime *J*_s_. The obtained minimum threshold of VPD driving force was consistent with results reported by Moore GW (2008) and Sellin A (2010) [42–43]. Both researchers suggested nighttime sap flow was elevated under relatively high *T*_a_ and VPD. There studies were carried out on *Tamarix elongata*, and *Juniperus scopulorum* in the semiarid regions where optimal VPD values reached 1.5 KPa and 2.5 KPa, respectively.

## Conclusions

Sap flow drives the physiological responses of desert plants which also respond to changes in the environmental variables. The sap flow of *Hedysarum scoparium* accelerated significantly under increasing evaporative demand, and refill water during the nighttime. The daily sap flow rate had more close correlation with reference evapotranspiration than with soil moisture content. Between day and night time, the sap flow rate responded to meteorological factors differently, and the intrinsic differences in physiology between different stems of *Hedysarum scoparium* may lead to differences in responding to meteorological factors. By using Redundancy analysis and the optimal regression model of the daily sap flow rate with the meteorological factors during the growing season that could be used to estimate the transpiration of *Hedysarum scoparium*.

## Supporting Information

S1 Dataset The excel “S3” has showed all the data we used in this paper, including sap flow, environmental variables and physiology characteristics related to this paper. Worksheets are arranged in the order of the corresponding Figs appears. Every worksheet has been named clearly and label in each rank is marked.
(XLSX)

S1 Fig The phenological change of *H*. *scoparium* (1^st^ May–15^th^ October 2011) (a represents new leaves appearing period; b represents flowers blooming period; c represents leaves withering period). (TIF)

S2 Fig Installation of the sap flow gauge on the stem of *H*. *scoparium* (a), and data logger description (b). (TIF)

S3 Fig The seasonal variation of sap flow rate on diameter of sample shrubs (1^st^ May–15^th^ October 2011). (TIF)

S4 Fig The seasonal variation of leaf area index between different stems of *H*.*scoparium* (1^st^ May–15^th^ October 2011). (TIF)
