# Supplementary figures and images for: The Sap Flow Dynamics and Response of Hedysarum scoparium to Environmental Factors in Semiarid Northwestern China
Source: PLoS One. 2015 Jul 2;10(7):e0131683. doi: 10.1371/journal.pone.0131683 (PMC4489904; doi:10.1371/journal.pone.0131683)

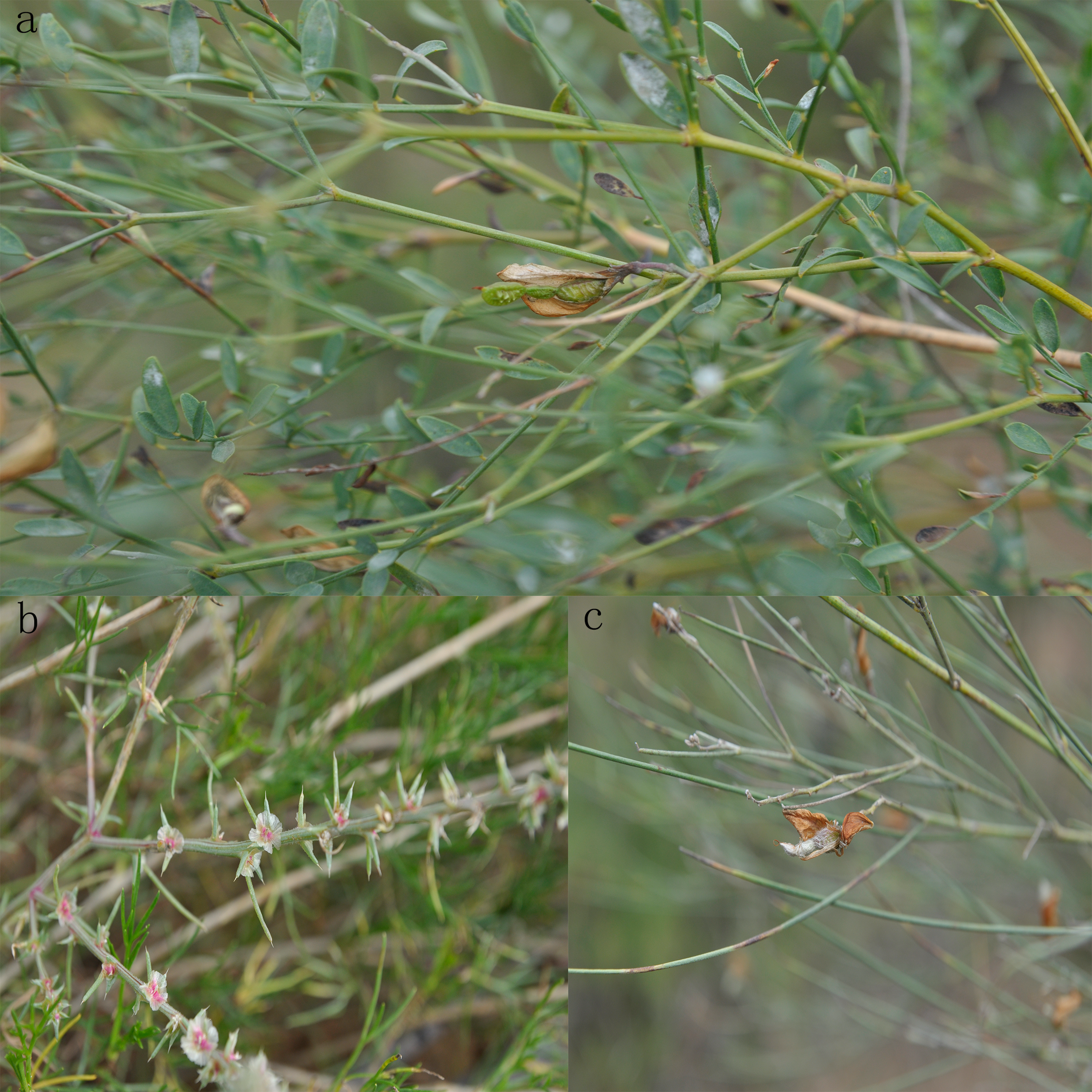

Supplement: S1 Fig — (TIF) [file pone.0131683.s002.tif]

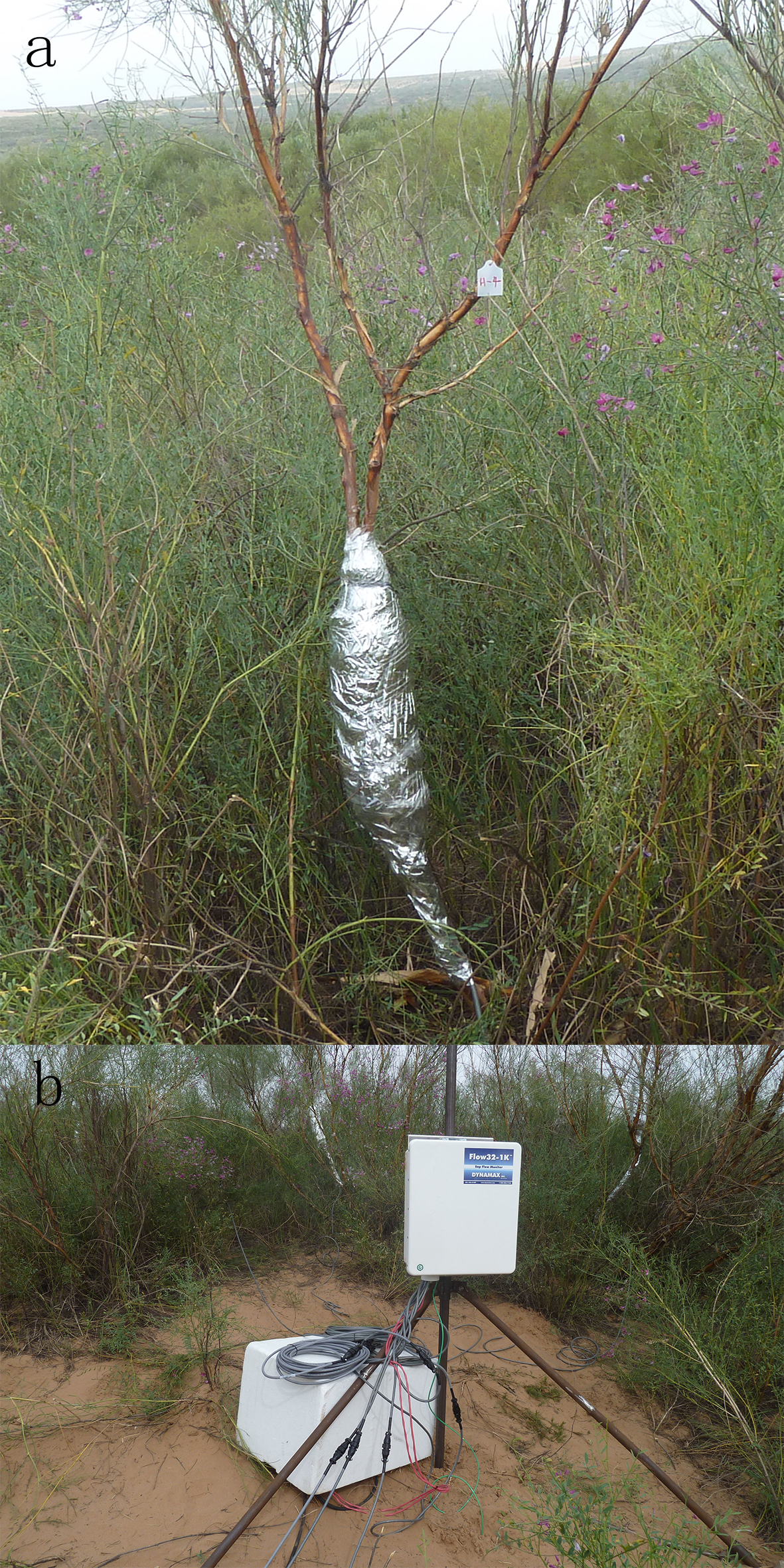

Supplement: S2 Fig — (TIF) [file pone.0131683.s003.tif]

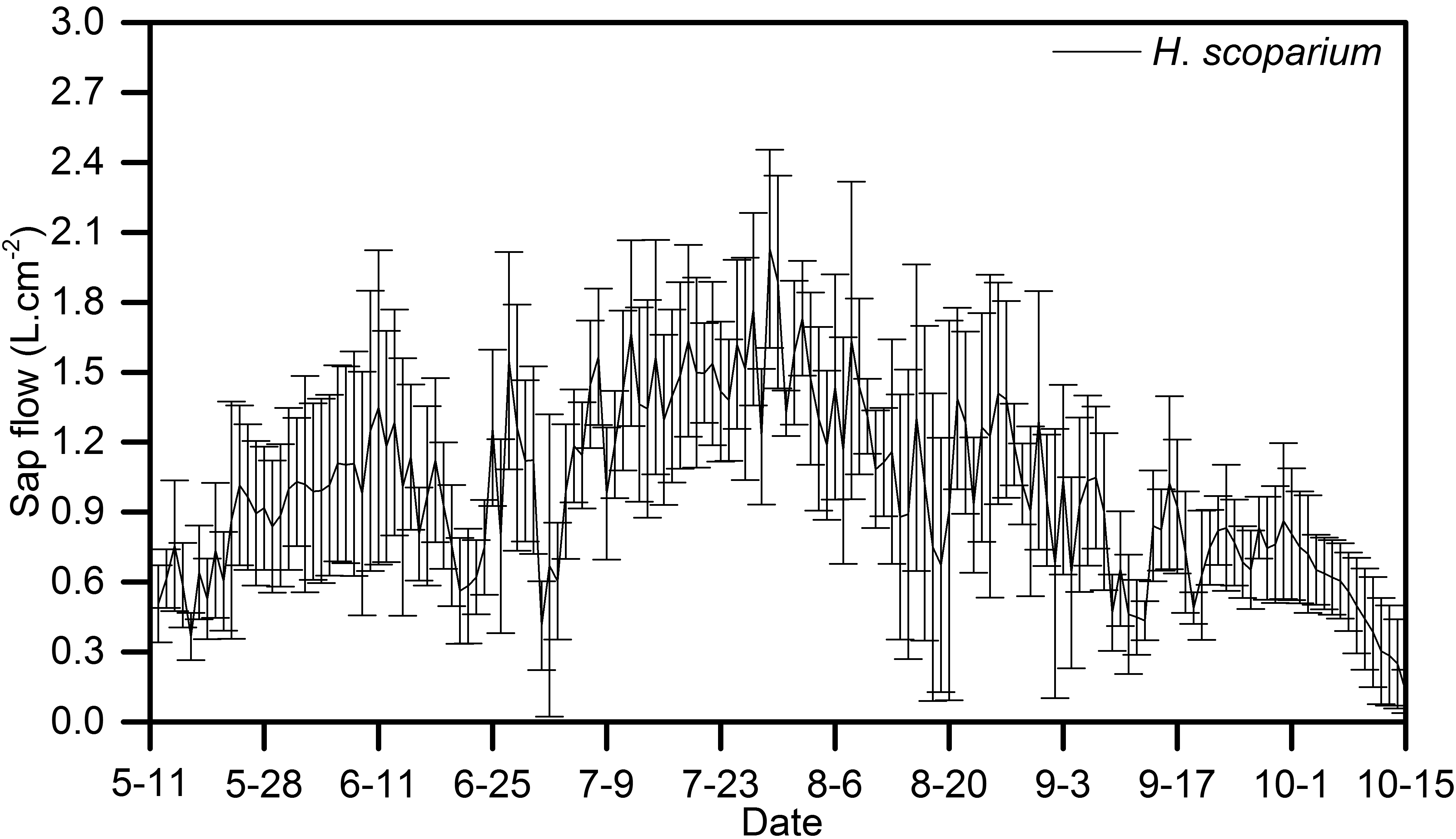

Supplement: S3 Fig — (TIF) [file pone.0131683.s004.tif]

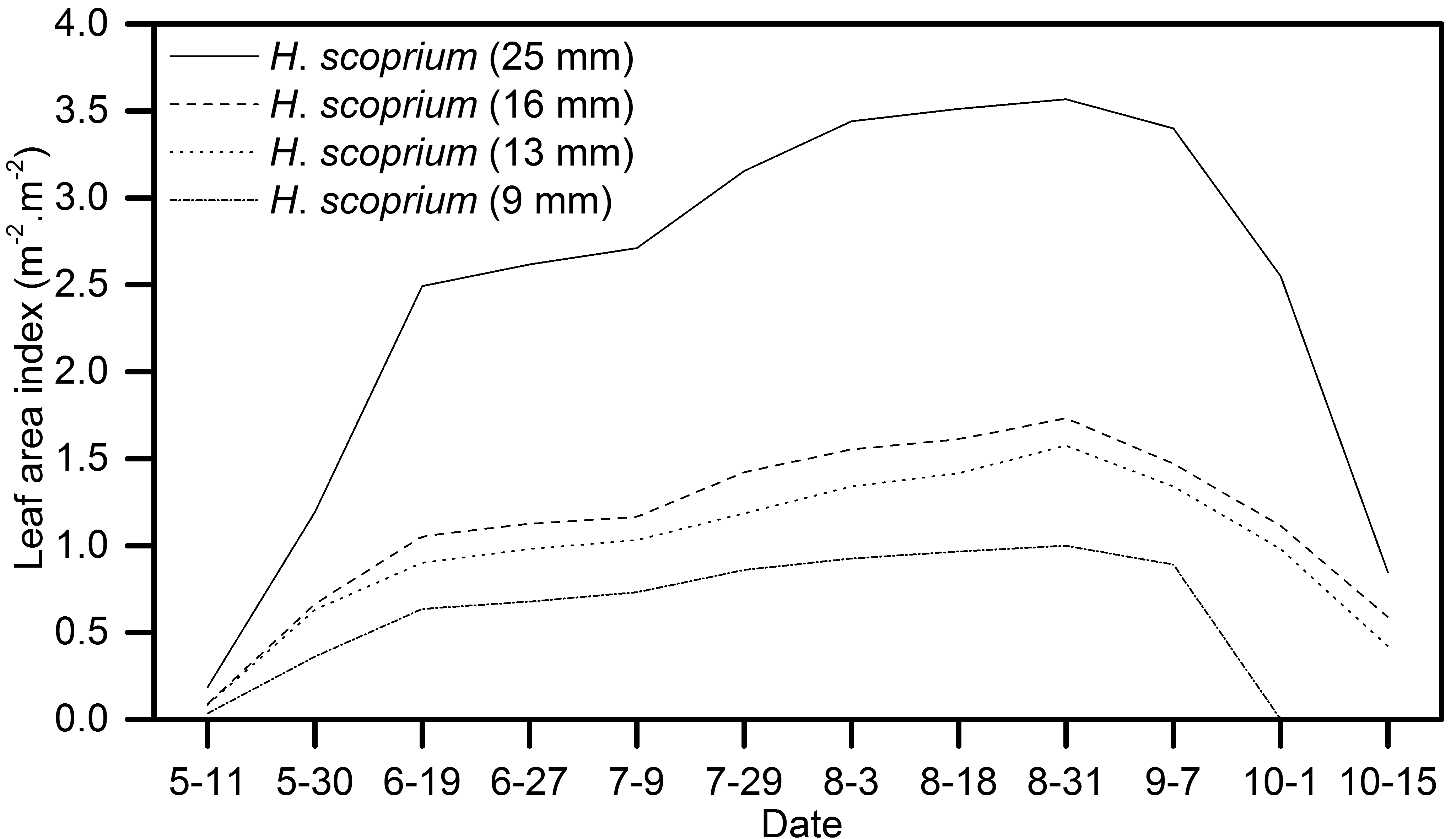

Supplement: S4 Fig — (TIF) [file pone.0131683.s005.tif]
